# Angiopoietin-like protein 4 dysregulation in kidney diseases: a promising biomarker and therapeutic target

**DOI:** 10.3389/fphar.2024.1475198

**Published:** 2025-01-07

**Authors:** Yan Li, Yuxin Zhang, Mengxia Cao, Tingting Yuan, Santao Ou

**Affiliations:** ^1^ Department of Nephrology, The Affiliated Hospital of Southwest Medical University, Luzhou, China; ^2^ Sichuan Clinical Research Center for Nephrology, Luzhou, China; ^3^ Metabolic Vascular Disease Key Laboratory of Sichuan Province, Luzhou, China

**Keywords:** angiopoietin-like protein 4, renal diseases, biomarker, therapeutics, mechanism, inflammation, oxidative stress

## Abstract

The global burden of renal diseases is increasingly severe, underscoring the need for in-depth exploration of the molecular mechanisms underlying renal disease progression and the development of potential novel biomarkers or therapeutic targets. Angiopoietin-like protein 4 (ANGPTL4) is a multifunctional cytokine involved in the regulation of key biological processes, such as glucose and lipid metabolism, inflammation, vascular permeability, and angiogenesis, all of which play crucial roles in the pathogenesis of kidney diseases. Over the past 2 decades, ANGPTL4 has been regarded as playing a pivotal role in the progression of various kidney diseases, prompting significant interest from the scientific community regarding its potential clinical utility in renal disorders. This review synthesizes the available literature, provides a concise overview of the molecular biological effects of ANGPTL4, and highlights its relationship with multiple renal diseases and recent research advancements. These findings underscore the important gaps that warrant further investigation to develop novel targets for the prediction or treatment of various renal diseases.

## 1 Introduction

The global burden of kidney diseases is immense, contributing directly to worldwide morbidity and mortality and serving as a significant risk factor for cardiovascular diseases. According to recent epidemiological data, approximately 1.2 million deaths annually are attributed to chronic kidney disease (CKD), and by 2040, CKD is projected to become the fifth leading cause of death globally (The Lancet, 2018; The Lancet, 2020). Additionally, other kidney disorders, such as nephrotic syndrome, diabetic kidney disease (DKD), acute kidney injury (AKI), and renal tumors, further exacerbate the overall burden. The medical burden on end-stage renal disease patients is particularly severe, necessitating long-term renal replacement therapy, with poor prognosis and high mortality rates imposing substantial economic pressure on public health systems.

However, current therapeutic strategies predominantly focus on managing symptoms and slowing disease progression rather than targeting the underlying pathophysiological mechanisms. Given the diversity of kidney diseases and the limitations of existing diagnostic and treatment approaches, it is imperative to explore novel early detection biomarkers and therapeutic targets.

Angiopoietin-like protein 4 (ANGPTL4) was initially reported by three independent research groups in 2000 and was identified through various methods, including the screening of novel downstream targets of peroxisome proliferator-activated receptors (PPARs), the identification of new fasting-induced factors from the liver, and the PCR-based discovery of novel angiopoietin-related proteins (Kersten et al., 2000; Kim et al., 2000; Yoon et al., 2000). ANGPTL4 is a member of the angiogenesis-regulating and secretory protein superfamily, known as the ANGPTL1--8 family, which encompasses eight types of secreted glycoproteins. With the exception of ANGPTL5, all these genes have been identified in both humans and mice (Zhu et al., 2012). The human ANGPTL4 gene is highly conserved across species, with 77% and 99% amino acid sequence homology with that of mouse and chimpanzee, respectively (Zhu et al., 2002). Located on chromosome 19p13.3, this gene consists of seven exons and encodes a glycosylated secretory protein (fANGPTL4). This protein is subsequently cleaved after rapid translation, producing an N-terminal coiled-coil domain (nANGPTL4) and a C-terminal fibrinogen-like domain (cANGPTL4) (Zhu et al., 2012). Alternative splicing generates multiple transcript variants. Its complex structure and regulatory systems enable ANGPTL4 to participate in various biological functions but also lead to different roles under diverse pathological conditions (Liu and He, 2019). Currently, ANGPTL4 shows significant potential as a prognostic or predictive biomarker and as a therapeutic target for various diseases.

In kidney diseases, the expression and function of ANGPTL4 are particularly significant, especially in conditions such as nephrotic syndrome, DKD, lupus nephritis (LN), renal cell carcinoma (RCC), dyslipidemia-induced renal damage, and AKI. Numerous studies have elucidated the potential roles of ANGPTL4 in these renal disorders, underscoring the need for a systematic review and analysis of existing research to better understand its role. This paper provides a comprehensive summary of recent advancements in the understanding of ANGPTL4 and consolidates the existing data on its role in kidney diseases.

## 2 Characteristics of ANGPTL4

### 2.1 Structure of ANGPTL4

**FIGURE 1:**
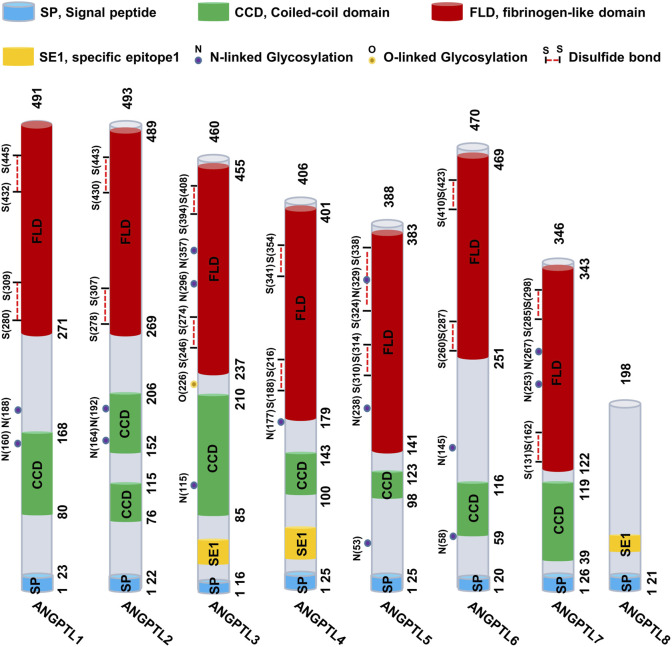
Schematic representation of the ANGPTL1-8 structure. The ANGPTL family typically comprises three conserved structural domains: the signal peptide (SP, blue), the coiled-coil domain (CCD, green), and the fibrinogen-like domain (FLD, red). However, ANGPTL8 is an exception, as it lacks the C-terminal FLD.

Angiopoietin-like proteins (ANGPTLs) constitute a family of secreted glycoproteins comprising eight members (ANGPTL1--8). These proteins share three conserved structural domains: a signal peptide (SP), a coiled-coil domain (CCD), and a fibrinogen-like domain (FLD). The only exception is ANGPTL8, which lacks the C-terminal FLD. (Figure 1).

**FIGURE 2:**
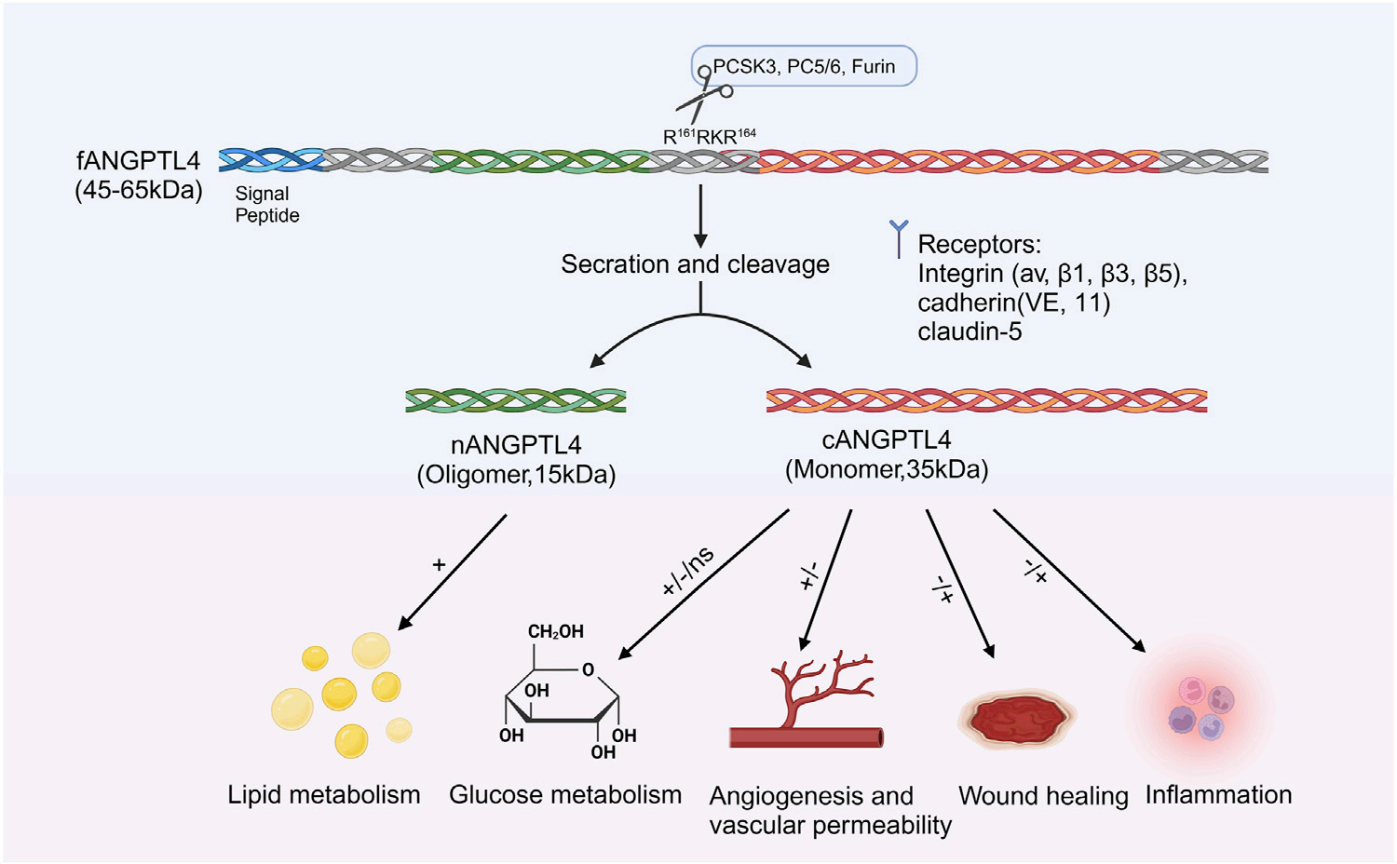
Structure and function of ANGPTL4. ANGPTL4 is composed of several distinct regions: the signal peptide, the N-terminal coiled-coil domain (nANGPTL4, 15 kDa), the linker region, and the C-terminal fibrinogen-like domain (cANGPTL4, 35 kDa). The glycosylated secretory protein (fANGPTL4, 45–65 kDa) undergoes proteolytic cleavage by proprotein convertases (including PCSK3, Furin, and PC5/6) at the amino acid sequence R161RKR164, as indicated by the scissor symbol. After cleavage, the ANGPTL4 domains are released from the cell, with nANGPTL4 remaining in an oligomeric state and cANGPTL4 dissociating into monomers. nANGPTL4 plays a crucial role in lipid metabolism, whereas cANGPTL4 is involved in several nonlipid-related processes. Created with BioRender.com.

ANGPTL4 consists of a secretory SP, an N-terminal CCD (nANGPTL4, 15 kDa), a linker region, and a C-terminal FLD (cANGPTL4, 35 kDa). (Ge et al., 2004; Zuo et al., 2023). The full-length ANGPTL4 (fANGPTL4, 45–65 kDa) undergoes proteolytic cleavage at R^161^RKR^164^ by proprotein convertases such as PCSK3, forming oligomeric nANGPTL4 and monomeric cANGPTL4 (Zhu et al., 2012; Fernández-Hernando and Suárez, 2020) (Figure 2). Before cleavage, fANGPTL4 forms higher-order structures via disulfide bonds. The cleavage patterns are tissue specific; adipose tissue secretes fANGPTL4, while the liver releases cleaved nANGPTL4. This structural complexity and tissue-specific expression highlight the diverse biological roles of ANGPTL4 (Zhu et al., 2012). Thus, the complex structure of ANGPTL4, along with its tissue-specific expression and cleavage patterns, underscores its multifaceted roles in various biological processes.

ANGPTL4 shares structural homology with angiopoietins but does not bind to their ligands Tie1 or Tie2 (Zhu et al., 2012), initially classifying it as an orphan receptor. Subsequent research identified integrins β1, β5, αvβ3, and α5β1 as key receptors, with ANGPTL4 also interacting with VE-cadherin and claudin-5 to regulate endothelial junction integrity (Goh et al., 2010; Huang et al., 2011; Gomez Perdiguero et al., 2016). In scar tissue, ANGPTL4 binds cadherin-11, reducing collagen expression via DNA-binding inhibitor 3 (Teo et al., 2017). It forms a complex with heparan sulfate and the Wnt coreceptor LRP6, mediating intracellular signaling (Kirsch et al., 2017). ANGPTL4 also binds neurofibromin 1/2 in endothelial cells, contributing to diabetic macular edema (Sodhi et al., 2019), and interacts with epidermal growth factor receptor, inhibiting granulosa cell proliferation in polycystic ovary syndrome (Jiang et al., 2023). Thus, ongoing research into ANGPTL4 receptors has expanded, offering new therapeutic opportunities. Targeting these specific receptor interactions can modulate the various physiological and pathological effects of ANGPTL4, providing new treatment strategies for fibrosis, vascular diseases, and metabolic disorders.

### 2.2 Functions of ANGPTL4

ANGPTL4 regulates various biological functions through its N-terminal and C-terminal domains. nANGPTL4, in particular, inhibits lipoprotein lipase (LPL) activity, leading to elevated circulating triglyceride (TG) levels (Fernández-Hernando and Suárez, 2020; Kersten, 2021). Desai et al. reported that ANGPTL4-deficient mice presented increased LPL activity, enhanced TG clearance, and reduced plasma TG levels (Desai et al., 2007). ANGPTL4 monoclonal antibodies decreased TG levels in high-fat diet-fed mice and monkeys (Dewey et al., 2016). Genetic studies have also shown that ANGPTL4 loss-of-function variants lower TG levels and increase HDL levels (Yin et al., 2009).

ANGPTL4 is involved in energy expenditure and diverse nonlipid processes, such as glucose metabolism, angiogenesis, vascular permeability, wound healing, inflammation, and oxidative stress (Figure 1). Studies on ANGPTL4 overexpression in mice have reported various effects on glucose metabolism, ranging from no impact to improved or impaired glucose tolerance, likely due to differences in the site and extent of overexpression (Xu et al., 2005; Mandard et al., 2006; Wang et al., 2016; Okamoto et al., 2017). In contrast, ANGPTL4 deficiency in adipose tissue enhances glucose tolerance in fat-specific knockout mice fed a high-fat diet (Aryal et al., 2018; Singh et al., 2018). In colorectal cancer, ANGPTL4 regulates glucose transporter expression, promoting glucose metabolism (Mizuno et al., 2022).

In recent years, the role of ANGPTL4 in regulating angiogenesis and vascular permeability has been controversial. It has been shown to inhibit vascular permeability and angiogenesis (Okochi-Takada et al., 2014; Gomez Perdiguero et al., 2016; Liabotis et al., 2022), yet it also promotes angiogenesis and disrupts barrier stability (Chong et al., 2014; Sodhi et al., 2019; Qiu et al., 2021). These apparent discrepancies may stem from differences in animal models, *in vitro* models, and experimental methods, with most studies focusing on the paracrine effects of ANGPTL4 on endothelial cells. Recently, Chaube et al. (2023) suggested considering the potential endothelial cell-specific autocrine effects of ANGPTL4. Local injection of recombinant ANGPTL4 can accelerate wound healing in diabetic mice (Chong et al., 2014). In contrast, another study revealed that in high-glucose-induced fibroblasts and diabetic mouse skin, IL-7 stimulates fibroblasts to secrete ANGPTL4, leading to delayed wound healing (Gao et al., 2023).

ANGPTL4 has both anti-inflammatory and proinflammatory effects. In colitis models, it stabilizes chemokine transcripts to protect against inflammation (Phua et al., 2017), whereas in psoriasis, it can promote inflammatory responses through ERK1/2 and STAT3 signaling (Zuo et al., 2022). During the early stages of oral inflammation, ANGPTL4 promotes the elevation of inflammatory factors, whereas in the later stages, excessive ANGPTL4 production exerts anti-inflammatory effects (Tian et al., 2022). These dual roles underline its complex regulation and tissue-specific functions. Given its broad involvement in metabolic disorders, inflammation, and cancer, ANGPTL4 has potential as a therapeutic target, although further research is needed to clarify its diverse mechanisms.

### 2.3 Modulators of ANGPTL4

In mice, ANGPTL4 is expressed primarily in adipose tissue, with lower levels in the heart, liver, and other tissues, whereas in humans, it is produced predominantly in the liver, adipose tissue, plasma, and heart (Kersten et al., 2000; Zuo et al., 2023). Its expression is regulated by nutritional status (such as fasting and caloric restriction) and metabolic conditions (such as hypoxia) (Hato et al., 2008; Zuo et al., 2023). ANGPTL4 levels significantly increase in human and mouse adipose tissue and plasma following fasting, which is likely mediated by changes in plasma insulin, cortisol, and fatty acids (Koliwad et al., 2009; Ruppert et al., 2020). Hypoxic stimulation also elevates ANGPTL4 expression in adipocytes and cancer cells (González-Muniesa et al., 2011; Drager et al., 2013; Niu et al., 2020; Tang et al., 2022; Zhang et al., 2022).

**FIGURE 3:**
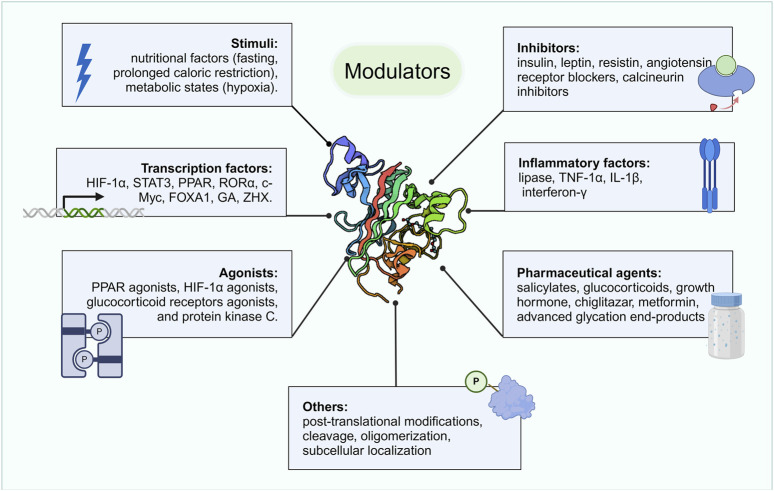
Modulators of ANGPTL4. ANGPTL4 expression is regulated by various factors, including specific nutritional and metabolic states, transcription factors, certain activators and inhibitors, inflammatory molecules, drugs, and posttranslational modifications. Created with BioRender.com.

In addition to the aforementioned stimuli, several studies have indicated that ANGPTL4 expression is regulated by specific activators and inhibitors, certain transcription factors, inflammatory molecules, and drugs (Figure 3). The key transcription factors that directly activate ANGPTL4 expression include hypoxia-inducible factor 1-alpha (HIF-1α) (Yang et al., 2019; Kang et al., 2021; Qi et al., 2023), signal transducer and activator of transcription 3 (STAT3) (Li et al., 2015; Avalle et al., 2022), PPARs (Alex et al., 2013; Cheng et al., 2021; Liu et al., 2023), retinoic acid receptor-related orphan receptor alpha (RORα) (Cho et al., 2019), c-Myc (Katanasaka et al., 2013), forkhead box A1 (FOXA1) (Mi et al., 2019), GA (Mi et al., 2019), and zinc finger and homeobox (ZHX) (Macé et al., 2020; Chugh and Clement, 2023). ANGPTL4 is also regulated by various agonists (such as PPAR and HIF-1α) and inhibitors (such as angiotensin blockers and insulin) (Grootaert et al., 2012; Shen et al., 2020; Zuo et al., 2023). Additionally, certain inflammatory factors (including lipases, TNF-α and others (Lu et al., 2010; Makoveichuk et al., 2017)) as well as various drugs (such as paeoniflorin, glucocorticoids and others (Chen et al., 2017; Lu et al., 2017; Pan et al., 2017; Yang et al., 2018; Hjelholt et al., 2020; Kang et al., 2020)) can modulate ANGPTL4 expression. Posttranslational modifications (such as sialylation, phosphorylation), cleavage, and subcellular localization further influence ANGPTL4 stability and function, increasing regulatory complexity (Zuo et al., 2023). Overall, the regulation of ANGPTL4 expression is a multifaceted process influenced by nutritional, metabolic, and environmental factors.

## 3 Role of ANGPTL4 in renal diseases

To date, extensive research has demonstrated that ANGPTL4 is aberrantly expressed in various kidney diseases and is closely related to the progression of these conditions through multiple mechanisms. This review summarizes the abnormal expression of ANGPTL4 in various kidney diseases, its specific mechanisms, and its pathogenic functions (Table 1).

**TABLE 1:** Roles of ANGPTL4 in renal diseases.

Diseases	Model	Dysregulation of ANGPTL4 levels	Intervention or pharmaceutical agents	Mechanisms and functions	References
Primary nephrotic syndrome	Lipopolysaccharide-induced mice	Upregulation in the renal tissue in the model group	Deletion of the ANGPTL4 gene	ANGPTL4 may induce mice podocyte injury and proteinuria formation by downregulating ACTN4 and podocin, while it is alleviated in ANGPTL4−/− mice	Li et al. (2023c)
	Adriamycin-induced mice	Upregulation in the renal tissue in the model group	Epigallocatechin-3-Gallate, YC-1	By suppressing the expression of HIF-1α, thereby resulting in a reduction of ANGPTL4 levels, mitigating oxidative stress and podocyte apoptosis, ameliorates renal function and structural damage	Liu and He (2019)
	Adriamycin-induced rats and podocyte	Upregulation in the model group within the renal cortex and podocytes	Paeoniflorin	Activate PPARγ to downregulate ANGPTL4, thereby significantly alleviating podocyte apoptosis, upregulating synaptic proteins, and reducing extracellular matrix to ameliorate podocyte injury	Lu et al. (2017)
	Adriamycin-induced rats and podocyte	Upregulation in the model group within the renal cortex and urine, as well as in podocytes	Salvianolic acid A+ low-dose prednisone	By facilitating the regulation of PPARγ, thereby attenuating the expression of ANGPTL4, a substantial reduction in podocyte injury and proteinuria is achieved	Wang et al. (2019)
	Puromycin aminonucleoside (PAN)-induced rats and podocyte	Upregulation in the model group within the renal cortex and podocytes	Calcineurin inhibitors, overexpression and downregulation of ANGPTL4	Targeting the NFATc1 pathway to decrease ANGPTL4 facilitates the restoration of PAN-induced synaptopathy reduction and podocyte apoptosis, mitigating podocyte injury	Shen et al. (2020)
	PAN-induced rat model	Upregulation in the model group within the renal cortex	PPAR γ agonist	By augmenting podocyte VEGF expression and diminishing ANGPTL4 to safeguard glomerular capillaries	Yang et al. (2006)
	Podocyte-specific overexpression of ANGPTL4 in transgenic rats and PAN-induced rats	Upregulation within the glomerulus, without a concomitant elevation in the systemic circulation	Podocyte-specific overexpression of ANGPTL4	The ANGPTL4 secreted by podocytes lacks proper sialylation, and hypo-sialylated ANGPTL4 may enhance its binding to the GBM, leading to exacerbation of renal disease and a substantial increase in proteinuria	Clement et al. (2011)
	Adipose tissue-specific overexpression of ANGPTL4 in transgenic rats and PAN-induced rats	Elevation in the systemic circulation	Adipose tissue-specific overexpression of ANGPTL4	ANGPTL4 within the circulatory system mitigates proteinuria by interacting with glomerular endothelial αvβ5 integrin. However, the circulatory ANGPTL4 impedes LPL, resulting in hypertriglyceridemia	Clement et al. (2014)
Diabetic kidney disease	STZ-induced rats	Upregulation in the model group within glomeruli and urine	—	The expression of ANGPTL4 in renal glomeruli is intricately associated with the levels of urinary albumin-to-creatinine ratio and podocyte injury. The urinary expression of ANGPTL4 is closely correlated with albuminuria in rat models	Ma et al. (2015)
	Mesangial cells stimulated by high glucose (HG)	Upregulation	Downregulation of ANGPTL4	Inhibiting the activation of the NF-κB signaling pathway significantly attenuated the proliferation of mesangial cells induced by HG, mitigated inflammatory responses, and curtailed extracellular matrix accumulation	Qin et al. (2019)
	Spontaneous diabetes in db/db mice and podocyte MPC5 cells treated with palmitic acid	Upregulation in the model group within the renal tissue and podocytes	Diosgenin, agonist of SIRT6	Inhibiting ANGPTL4 could emerge as a pivotal determinant in safeguarding podocyte injury downstream of SIRT6	Wang et al. (2022)
	STZ-induced mice and HG-cultured MPC-5	Upregulation in the model group within the renal glomerulus and podocytes	ManNAc, overexpression of ANGPTL4	Modulating the ROS/NLRP3 signaling pathway to counteract the protective effect of ManNAc on podocyte injury	Gao et al. (2022)
	STZ-induced rat model and MPC-5 stimulated by HG.	Upregulation in the model group within the renal cortex and podocytes	ANGPTL4-neutralizing antibody	By mitigating the activation of the integrin-β1/FAK signaling pathway, alleviating podocyte apoptosis, and ameliorating the disruption of the actin cytoskeleton	Gao et al. (2022)
Lupus nephritis	Female MRL/LPR mice prone to lupus	Upregulation in the MRL/LPR mice within the renal tissue	Deletion of the ANGPTL4 gene	By inhibiting the NLRP3 inflammasome to suppress the inflammatory response, the generation of pro-inflammatory cytokines in the renal milieu is restrained	Luo et al. (2023)
Renal cell carcinoma	786-O and Caki cell lines	Upregulation	Downregulation of ANGPTL4	By attenuating the phosphorylation levels of p38 and ERK proteins, the cell cycle is protracted delayed, concurrently diminishing clonogenicity and proliferative capacity	Ma et al. (2023a)
	CAKI-1 cell line	Upregulation	Overexpression of HM1-3 subtypes	Upregulation of ANGPTL4 mRNA in CAKI-1 cells subjected to HOTAIRM1 knockdown	Hamilton et al. (2020)
	The CAKI-1 cell line with cobalt chloride treatment	Upregulation	SiRNA targeting HIF1α or HIF1β	The knockout of HOTAIRM1 failed to induce ANGPTL4, suggesting the necessity of HIF1 signal transduction	Hamilton et al. (2020)
Hyperlipidemic renal injury	Mice subjected to a high-fat dietary regimen	Gradually upregulating in the model group, the glomeruli	Deletion of the ANGPTL4 gene	Modulating the expression of podocyte ACTN4, plays a pivotal role in the renal injury induced by hyperlipidemia	Li et al. (2022)
	Feeding rats a high-fat diet, stimulating human podocytes with palmitic acid	Gradually upregulating in the model group, the glomeruli	AICAR	Activation of the AMPK/ACC signaling pathway can downregulate the intracellular expression of ANGPTL4 in podocytes, thereby mitigating podocyte injury	Qiu et al. (2023)
Acute renal injury	Cisplatin-induced mice	Upregulation in the proximal tubules of the model group	PPARα ligand	Enhancing the expression of GPHBP1 and Lmf1 to augment LPL activity, and diminishing the expression of ANGPTL4 to ameliorate renal toxicity	Li et al. (2012)

### 3.1 ANGPTL4 and nephrotic syndrome

Nephrotic syndrome is a kidney disorder characterized by damage to the glomerular filtration barrier and an increased filtration rate, resulting in significant proteinuria, hypoalbuminemia, edema, hyperlipidemia (elevated triglycerides and cholesterol), and lipiduria (Chugh et al., 2012). Common primary causes of nephrotic syndrome include minimal change disease (MCD), focal segmental glomerulosclerosis (FSGS), and membranous nephropathy (MN), all of which are forms of primary glomerular disease (Clement et al., 2015). Despite extensive research identifying key structural proteins that may lead to glomerular filtration defects, many pathogenic mechanisms underlying nephrotic syndrome remain unclear.

Recent studies suggest that ANGPTL4 may play a crucial role in the pathogenic mechanisms of nephrotic syndrome (Clement et al., 2014; Chugh and Clement, 2023). In a lipopolysaccharide (LPS)-induced nephropathy model, ANGPTL4 gene knockout alleviated hyperlipidemia and proteinuria (Li et al., 2023c). This improvement is associated with the downregulation of the actin cytoskeleton regulatory factors ACTN4 and podocin. Similarly, ANGPTL4 levels are significantly elevated in rat models induced by puromycin aminonucleoside (PAN) and in podocyte injury models. Overexpression and knockdown experiments revealed that ANGPTL4 can directly induce podocyte cytoskeletal rearrangement, reduce synaptopodin expression, and exacerbate PAN-induced podocyte apoptosis (Yang et al., 2006; Shen et al., 2020). Further research has shown that ANGPTL4 expression is significantly upregulated following doxorubicin treatment, whereas epigallocatechin gallate (EGCG) may alleviate FSGS by targeting the HIF-1α/ANGPTL4 pathway to inhibit oxidative stress and podocyte apoptosis (Liu and He, 2019). Downregulation of ANGPTL4 reduces cell apoptosis, improves podocyte injury, and decreases proteinuria, a process regulated by PPARγ (Lu et al., 2017; Wang et al., 2019). These findings collectively underscore the critical role of ANGPTL4 in the progression of nephrotic syndrome, particularly through its impact on podocyte structure and function and its involvement in disease pathogenesis. However, it is important to note that most existing research focuses on animal models and cellular studies, with a lack of validation in human subjects. Therefore, the potential mechanisms by which ANGPTL4 is involved in nephrotic syndrome require further investigation.

In clinical studies, increased serum ANGPTL4 levels have been observed in patients with MCD and MN compared with healthy controls, and serum ANGPTL4 levels are closely correlated with proteinuria and renal function in MCD patients (Shen et al., 2020). These findings suggest that ANGPTL4 may play a significant role in the pathological processes of these kidney diseases, especially when renal filtration barrier function is impaired. Surprisingly, another study revealed no significant associations between ANGPTL4 levels in blood or urine and proteinuria in MCD patients (Cara-Fuentes et al., 2017). Additionally, this study revealed that patients with severe proteinuria, regardless of whether they had MCD, FSGS, or MN, had significantly increased urinary excretion of ANGPTL4, with levels remaining elevated throughout the disease process (Cara-Fuentes et al., 2017). The discrepancies in these findings may stem from variations in sample size, individual differences, and experimental reagents. Future large-scale, multicenter clinical studies are needed to comprehensively assess the effectiveness and specificity of ANGPTL4 as a biomarker for nephrotic syndrome in blood and urine. These studies should consider different types of kidney diseases, disease stages, and patient variability and use standardized detection methods and criteria as much as possible.

Chugh and colleagues recently made a groundbreaking discovery emphasizing the critical role of low-sialylated ANGPTL4 in podocytes in understanding the major manifestations of human MCD (Chugh and Clement, 2023). They reported that podocytes secrete two distinct forms of ANGPTL4: one sialylated form with a neutral isoelectric point (pI) and another high pI form lacking sialic acid residues (lowly sialylated ANGPTL4), which is observed exclusively in the glomeruli and urine (Chugh and Clement, 2023). In studies involving glomerular endothelial cells cultured under oxidative stress, sialylated ANGPTL4 significantly reduced cell damage, whereas low-sialylated ANGPTL4 exacerbated damage (Clement et al., 2014). Furthermore, rats overexpressing ANGPTL4 specifically in podocytes presented high pI and low ANGPTL4 sialylation, accompanied by pronounced proteinuria, loss of glomerular basement membrane charge, and effacement of podocyte foot processes (Clement et al., 2011). These detrimental effects are attributed to the greater affinity of low-sialylated ANGPTL4 for integrins αvβ5 and α3β1 and its potential strong binding to proteoglycans such as transmembrane proteoglycans and basement membrane proteoglycans (Chugh and Clement, 2023).

Studies have shown that the nuclear factor ZHX1 significantly upregulates low-sialylated ANGPTL4, leading to glomerular injury associated with low sialylation (Clement et al., 2011). Additionally, in ANGPTL4 knockout mice, the administration of lipopolysaccharide or nephrotoxic serum significantly reduced proteinuria, whereas no significant difference was observed in the control groups (Clement et al., 2011). These findings indicate that low-sialylated ANGPTL4 secreted by podocytes plays a crucial role in nephrotic syndrome. Moreover, oral administration of low-dose N-acetyl-D-mannosamine (ManNAc) improved ANGPTL4 sialylation *in vivo* and significantly reduced proteinuria (Chugh et al., 2014). Therefore, sialic acid precursor therapy could be a potential treatment to reduce proteinuria in certain nephrotic syndromes. However, the long-term safety and efficacy of ManNAc or other sialic acid precursors as therapeutic agents for nephrotic syndrome need to be evaluated in future clinical trials.

Low sialylated ANGPTL4 is more likely to bind with glomeruli, while the majority of ANGPTL4 circulating in the bloodstream is a sialylated protein with a neutral pI secreted by peripheral tissues (adipose tissue, skeletal muscle, heart, and liver), with a smaller fraction of low sialylated ANGPTL4 also being secreted into the circulation by podocytes (Clement et al., 2011). The overexpression of adipose tissue-specific ANGPTL4 in transgenic rats results in increased circulating ANGPTL4 levels without accompanying proteinuria, highlighting the specificity and significance of podocyte-derived ANGPTL4 in proteinuric diseases. Analysis of ANGPTL4 oligomer formation in transgenic rats revealed that the majority of ANGPTL4 in the glomeruli is in the form of monomers or low-order oligomers, whereas circulating ANGPTL4 predominantly exists as intermediate- and high-order oligomers, which may account for why the circulating form of ANGPTL4 does not penetrate the glomerular basement membrane (Clement et al., 2011). The secretion of circulating ANGPTL4 occurs when proteinuria reaches the nephrotic threshold, with a negative feedback loop wherein circulating ANGPTL4 interacts with glomerular integrin αvβ5 to mitigate proteinuria. However, in minimal change disease (MCD), this feedback loop is compromised by the deleterious and overwhelming effects mediated by low sialylated ANGPTL4 (Chugh et al., 2014; Clement et al., 2014; Macé and Chugh, 2014).

**FIGURE 4:**
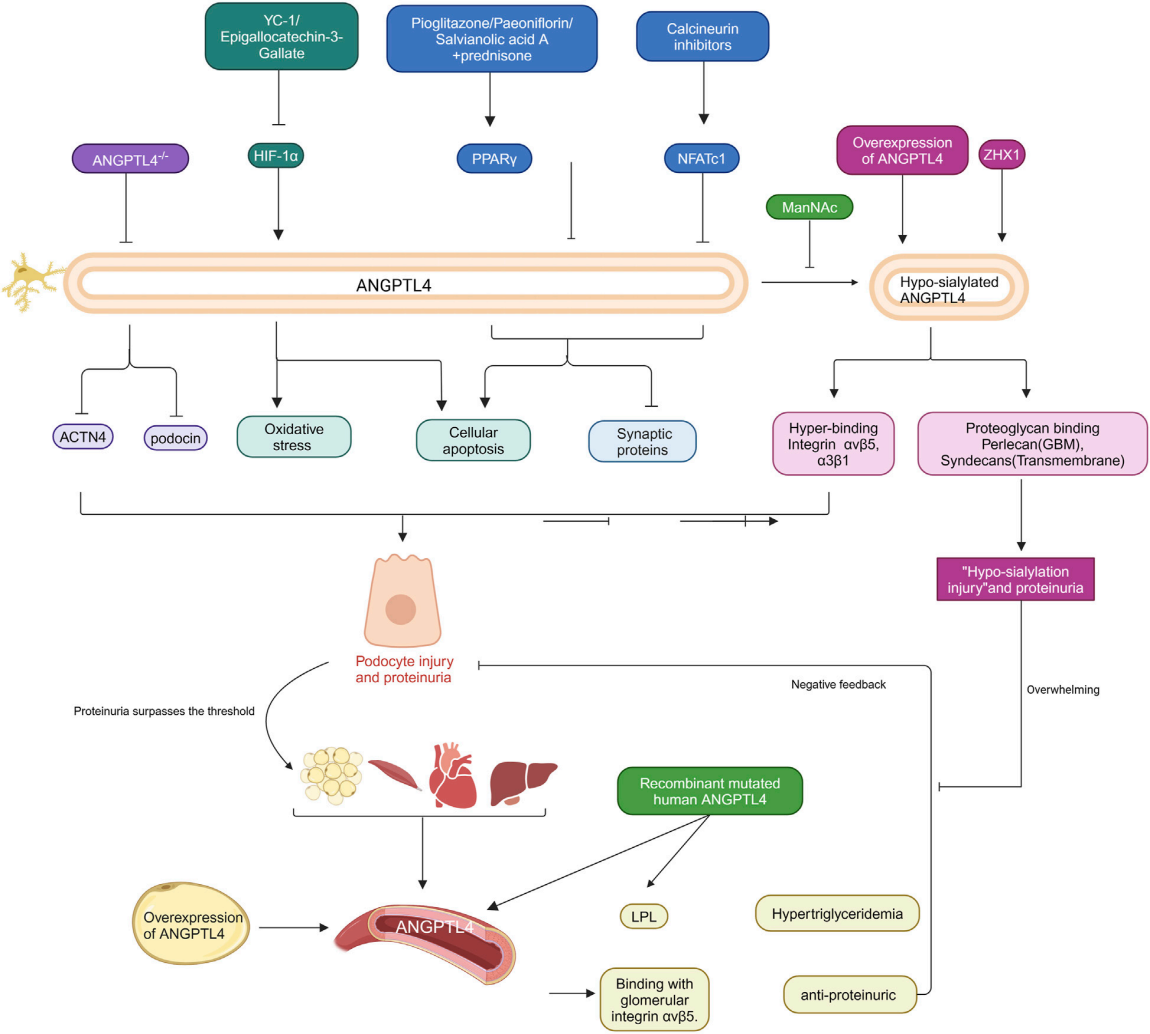
Role of ANGPTL4 in primary nephrotic syndrome. The specific regulatory mechanisms and intervention targets of podocytes and circulating ANGPTL4 in the pathogenesis of primary nephrotic syndrome are delineated. Podocyte-secreted ANGPTL4 modulates various cellular functions, including oxidative stress, apoptosis and cytoskeletal rearrangement. In contrast, circulating ANGPTL4 undergoes negative feedback regulation in response to substantial proteinuria. The intricate mechanistic actions of these ANGPTL4 entities are explained in the text. The arrows in the figure signify promoting effects, whereas the blocked arrows denote inhibitory effects. Created with BioRender.com.

Additionally, circulating ANGPTL4 induces hypertriglyceridemia by inhibiting LPL. Studies have shown that intravenous administration of recombinant mutant human ANGPTL4, which modifies key interaction sites with LPL, significantly reduces proteinuria in FSGS-afflicted animals without altering plasma TG levels (Clement et al., 2014; Del Nogal-Avila et al., 2016). Thus, recombinant mutant human ANGPTL4 is being developed as a promising therapeutic approach for nephropathy. In summary, the mechanistic role of ANGPTL4 in renal diseases, particularly proteinuric disorders, is complex and multifaceted. The low level of sialylated ANGPTL4 produced by podocytes plays a crucial role in the onset and progression of proteinuria, while circulating ANGPTL4 secreted by peripheral tissues affects lipid metabolism and glomerular function through different mechanisms. Future research should continue to explore the various forms of ANGPTL4 and their specific roles in nephropathy, with a particular focus on the clinical potential of sialic acid precursors and recombinant mutant ANGPTL4 as therapeutic strategies for nephrotic syndrome. Figure 4 summarizes the role of ANGPTL4 in primary nephrotic syndrome.

### 3.2 ANGPTL4 and diabetic kidney disease (DKD)

DKD is one of the most prevalent complications of diabetes and a leading cause of end-stage renal disease worldwide (Ogurtsova et al., 2017; Lv and Zhang, 2019; Saeedi et al., 2019). Pathologically, DKD is characterized by histological features, including glomerular and tubular basement membrane thickening, mesangial expansion, extracellular matrix accumulation, podocyte cytoskeletal rearrangement, and tubular cell injury (Remuzzi et al., 1997; Bose et al., 2017). Recent research has highlighted the significant potential of ANGPTL4 in the early diagnosis of DKD, particularly in detecting podocyte dysfunction, indicating promising application prospects.

In studies using streptozotocin (STZ)-induced diabetic rats and spontaneous diabetic db/db model mice, ANGPTL4 expression in renal tissues was found to be significantly elevated. Additionally, ANGPTL4 levels are increased in the urine of STZ-induced diabetic patients, with ANGPTL4 expression in renal tissues and urine closely correlated with urinary ALB levels (Ma et al., 2015; Wang et al., 2022). These findings suggest that ANGPTL4 may serve as a novel and potential diagnostic and therapeutic biomarker for DKD. *In vitro* studies evaluating ANGPTL4 under high glucose (HG) conditions revealed that high glucose stimulation significantly increased ANGPTL4 expression, whereas ANGPTL4 knockdown markedly inhibited HG-induced cell proliferation and inflammatory responses (Qin et al., 2019). Moreover, comprehensive bioinformatics analysis of transcriptomic data from widely used tetracycline and STZ-induced DKD models, with validation in db/db mice, indicated that ANGPTL4 could be a key gene in the pathogenesis of DKD (Xu et al., 2023), although its precise mechanisms warrant further investigation. Gao et al. (2022) reported that the upregulation of ANGPTL4 was associated with podocyte damage in DKD mice. Under HG conditions, ANGPTL4 overexpression *in vitro* counteracted the protective effects of ManNAc against podocyte injury via the ROS/NLRP3 signaling pathway. Similarly, ANGPTL4 upregulation activated the integrin-β1/FAK signaling pathway, promoting podocyte apoptosis and actin cytoskeletal disruption, which could be reversed by ANGPTL4-neutralizing antibodies or ManNAc supplementation (Guo et al., 2020). These findings suggest that targeting ANGPTL4-related signaling pathways has therapeutic potential for DKD. Future research should continue to explore the specific mechanisms of ANGPTL4 in diabetic kidney disease and develop ANGPTL4-based diagnostic and therapeutic strategies to provide more effective treatment options for DKD patients.

In clinical studies, circulating ANGPTL4 levels are significantly elevated in patients with DKD. Interestingly, this elevation is specific to the renal disease state, with comparable ANGPTL4 levels in type 2 diabetes patients and controls (Al Shawaf et al., 2019). Furthermore, serum ANGPTL4 levels positively correlate with clinical biomarkers of DKD (such as the urine albumin-to-creatinine ratio and serum creatinine) and negatively correlate with the estimated glomerular filtration rate (eGFR) (Al Shawaf et al., 2019), suggesting that circulating ANGPTL4 may serve as a biochemical marker for detecting renal disease status in diabetic patients. A single-center cross-sectional study by Bano et al. (2023) further supported this finding. Their research indicated that, compared with DKD patients with normal albuminuria, those with heavy albuminuria presented significantly higher levels of ANGPTL4 in the urine, which was negatively correlated with the eGFR and increased with worsening renal function. Even after adjusting for demographic, clinical, and laboratory parameters, urine ANGPTL4 levels remained significantly associated with DKD. Their study also demonstrated that urine ANGPTL4 performed exceptionally well in DKD diagnosis, with AUC values of 0.90 and 1.00 for the microalbuminuria and heavy albuminuria groups, respectively, and specificities of 93.3% and 97.8%, respectively. These findings suggest that urine ANGPTL4 expression could serve as a preliminary diagnostic marker for DKD and that its diagnostic efficacy may be enhanced when it is combined with other indicators. However, despite these encouraging findings, further longitudinal studies across diverse large ethnic populations are necessary to validate its applicability.

In summary, the role and potential of ANGPTL4 in DKD are progressively being revealed, with its expression levels in both circulation and urine significantly correlating with renal function indicators, demonstrating its potential as a diagnostic and prognostic marker. Future research should continue to explore the specific mechanisms of ANGPTL4, particularly in larger and more diverse populations, to provide more feasible and effective diagnostic and therapeutic strategies for DKD patients.

### 3.3 ANGPTL4 and lupus nephritis (LN)

Systemic lupus erythematosus (SLE) is a chronic, systemic autoimmune disorder characterized by heterogeneous clinical manifestations and multiorgan involvement (Tanaka, 2022). LN damage results from SLE and is one of the most severe organ manifestations of the disease, potentially progressing to end-stage renal disease in its late stages and serving as a major cause of mortality in SLE patients (Anders et al., 2020). In recent years, researchers have extensively studied biomarkers for LN as alternatives to renal biopsy, the gold standard (Soliman and Mohan, 2017; Vanarsa et al., 2020). These studies aim to facilitate more convenient longitudinal monitoring of patients, enabling close tracking of disease progression and timely adjustments to treatment regimens.

Vanarsa et al. (2020) employed a novel quantitative planar protein microarray to screen 1,000 proteins in the urine of SLE patients and reported that ANGPTL4 levels were significantly elevated in the urine of patients with active renal SLE compared with healthy controls and were markedly higher than those in patients with active nonrenal SLE. In the diagnosis of LN, urine ANGPTL4 demonstrated excellent performance, with an AUC of 0.96 and a specificity of 87.5%, suggesting that ANGPTL4 could serve as a diagnostic biomarker for LN in SLE patients. Additionally, during follow-up examinations of LN patients, urine ANGPTL4 was found to be present either prior to or concomitantly with worsening of the SLEDAI or rSLEDAI, indicating its reliability and significance as a clinical marker of disease activity and suggesting its utility as a novel urinary biomarker for tracking LN disease activity. However, urine ANGPTL4 requires further independent validation in larger cohorts. The specific mechanisms of ANGPTL4 in LN remain unclear. Recent studies have indicated that silencing ANGPTL4 significantly reduces urinary protein, creatinine, and urea nitrogen levels in MRL/LPR mice and improves renal pathological changes (Luo et al., 2023). This effect may be related to inactivation of the NLRP3 inflammasome, thereby suppressing inflammatory responses, such as the inhibition of TNF-α, IL-17, and monocyte chemoattractant protein-1, in MRL/LPR mice with lupus nephritis (Luo et al., 2023). These findings suggest that targeting ANGPTL4 expression may represent a viable new therapeutic approach for LN, but further validation is needed in other lupus nephritis animal models and clinical trials. In summary, the role of ANGPTL4 in SLE, particularly LN, is gaining increasing attention. Its expression levels in urine are closely associated with LN diagnosis and disease activity, demonstrating promising potential as a biomarker for both the diagnosis and monitoring of LN.

### 3.4 ANGPTL4 and renal cell carcinoma (RCC)

RCC is a malignancy originating from the renal tubular epithelium and accounts for more than 90% of kidney cancers. Among RCC subtypes, clear cell renal cell carcinoma (ccRCC) is the most prevalent and aggressive, accounting for 75% of RCC cases, and is associated with a high mortality rate (Hsieh et al., 2017). The pathogenesis of RCC is intricate and multifaceted, with a lack of distinctive clinical features in the early stages; consequently, 25%–30% of patients present with metastasis at diagnosis (Ljungberg, 2007). The risk of metastasis and recurrence remains high following local renal tumor resection, and RCC is resistant to chemotherapy and radiotherapy, leading to a poor prognosis (Cohen and McGovern, 2005; Wood, 2007). Thus, identifying biomarkers for early diagnosis and therapeutic targets is crucial for RCC.

ANGPTL4 is highly expressed in various cancers, including colorectal, prostate, breast, and liver cancers, where it plays roles in regulating tumor growth, angiogenesis, redox balance, tumor invasion, and metastasis (Ma B. et al., 2023). In ccRCC tumor tissues, ANGPTL4 expression is also significantly elevated (Le Jan et al., 2003; Verine et al., 2010), suggesting a potential role for ANGPTL4 in ccRCC. Moreover, serum ANGPTL4 levels are even higher in RCC patients than in patients with other solid tumors (Dong et al., 2017), indicating that ANGPTL4 may play a more specific role in the development and progression of kidney cancer. Previous studies identified ANGPTL4 mRNA expression as a diagnostic marker for both primary and metastatic RCC but lacked prognostic value (Verine et al., 2010). In contrast, other studies suggest that serum ANGPTL4 may serve not only as a diagnostic marker but also as a prognostic biomarker for RCC (Dong et al., 2017). This discrepancy might be due to differences between the mRNA and protein expression levels, which reflect various aspects of the disease and different detection methodologies. Future research should validate the diagnostic efficacy and prognostic value of serum ANGPTL4 in RCC patients within larger cohorts.

Recent advancements in bioinformatics have accelerated the screening and study of characteristic genes in RCC. For example, machine learning algorithms and databases have identified ANGPTL4 as a potential prognostic biomarker for ccRCC patients (Wang et al., 2018; Zhang et al., 2020; Han and Song, 2022; Li L. et al., 2023). In disease prognostic models, ANGPTL4 has demonstrated diagnostic potential, with an AUC of 0.7665, and its expression in RCC tumor samples is significantly greater than that in normal kidney tissue (Li L. et al., 2023). However, most of these studies remain at the bioinformatics level and lack validation from large clinical samples or *in vitro* and *in vivo* experiments. Apanovich et al. (2020) employed microarray expression databases to identify the top 20 genes expressed in ccRCC tumors and further examined these genes in 68 paired ccRCC tumor and normal samples. The results revealed that ANGPTL4 expression was elevated in stage I/II ccRCC samples compared with normal kidney tissue but was significantly reduced in stage IV samples, which may be associated with the expression levels of inducible HIF-1 genes (Zhang et al., 2013; Apanovich et al., 2021). Under hypoxic conditions during the development of stage I/II ccRCC tumors, ANGPTL4 might promote tumor progression by preventing apoptosis and modulating the redox shift toward ROS formation (Zhu et al., 2011; Baba et al., 2017).

Studies indicate the presence of multiple hypoxia-related tumor microenvironment cell subpopulations in ccRCC, with ANGPTL4+ endothelial cells potentially playing a pivotal role in tumor angiogenesis, indicating significant prognostic value. *In vitro* experiments suggest that the knockdown of ANGPTL4 may impact ccRCC cell proliferation by modulating the ERK/P38 signaling pathway (Ma B. et al., 2023). Additionally, knockdown of HOTAIRM1 lncRNA in CAKI-1 cells led to increased HIF-1α protein expression, thereby activating ANGPTL4 expression (Hamilton et al., 2020). These findings reveal that high ANGPTL4 expression in ccRCC may facilitate tumor growth and angiogenesis, while ANGPTL4 inhibition might suppress tumor cell proliferation by regulating key signaling pathways. In summary, through bioinformatics analysis and preliminary experimental validation, the role and potential of ANGPTL4 in RCC are becoming increasingly evident. Future research should delve deeper into the specific mechanisms of ANGPTL4 in RCC and validate its application value through larger clinical samples and comprehensive *in vitro* and *in vivo* experiments, providing new insights and methods for the early diagnosis and treatment of RCC.

### 3.5 ANGPTL4 and hyperlipidemia-induced renal injury

Dyslipidemia is a crucial marker of CKD progression, and dyslipidemia has been identified as an independent risk factor for CKD management (National Library of Medicine, 2002). Dyslipidemia adversely affects visceral organs, particularly the kidneys. Renal damage caused by dyslipidemia is related not only to lipotoxicity but also to oxidative stress, endoplasmic reticulum stress, and inflammatory responses (Chen et al., 2023). Since Moorhead et al. (1982) proposed the “lipid nephrotoxicity hypothesis” in 1982, accumulating evidence has supported the notion that lipid abnormalities can lead to glomerulosclerosis and interstitial kidney disease (Tomiyama-Hanayama et al., 2009; Liu, 2011). However, the specific mechanisms by which dyslipidemia significantly impacts the progression and development of renal damage remain inadequately understood.

ANGPTL4 is a key molecule involved in regulating lipid metabolism. Studies have shown that ANGPTL4 gene knockout mice exhibit significantly reduced TG levels and increased LPL activity (Adachi et al., 2009; Nyrén et al., 2019). In contrast, transgenic mice with high ANGPTL4 expression present markedly elevated TG levels (Clement et al., 2014), resulting in disrupted lipid metabolism. Compared with healthy controls, patients with dyslipidemic renal damage have significantly elevated ANGPTL4 levels in both the serum and urine. Serum ANGPTL4 levels are positively correlated with blood lipid levels, whereas urinary ANGPTL4 levels are positively correlated with urinary protein levels (Gao et al., 2020). However, whether serum and urinary ANGPTL4 levels independently influence blood lipid or urinary protein levels in dyslipidemic patients requires validation through larger cohort studies. Research on the mechanisms of ANGPTL4 in dyslipidemia-induced renal damage remains limited. Li et al. (2022) were the first to investigate ANGPTL4 expression in a high-fat renal injury animal model and reported that under high-fat conditions, ANGPTL4 expression in wild-type mouse renal tissues was significantly upregulated and increased progressively with increasing duration of feeding. ANGPTL4 gene knockout significantly reduced dyslipidemia, proteinuria, and podocyte foot process effacement in mice, potentially implicating ANGPTL4 in the pathogenesis of dyslipidemia-induced renal damage by affecting podocyte ACTN4 expression. Recent studies have shown that ANGPTL4 levels are markedly increased in the urine and renal tissues of rats fed a high-fat diet, and lipid accumulation in human podocytes treated with palmitic acid is accompanied by increased ANGPTL4, suggesting that ANGPTL4 may be a critical factor in dyslipidemic renal damage (Qiu et al., 2023). Further research revealed that activation of the AMPK/ACC signaling pathway can downregulate ANGPTL4 expression in podocytes, offering protective effects against saturated fatty acid-induced podocyte injury (Qiu et al., 2023). However, these results need further validation through additional experiments. Future studies should continue to explore the specific mechanisms of ANGPTL4 in dyslipidemia-induced renal damage and evaluate its potential as a novel biomarker for treating dyslipidemic kidney disease.

### 3.6 ANGPTL4 and other renal diseases

Acute decompensated heart failure (ADHF)-induced AKI studies in sheep models have identified ANGPTL4 as a potential candidate biomarker for long-term renal impairment associated with acute ADHF on the basis of transcriptomic analyses (Rademaker et al., 2021). These findings offer new insights into the molecular mechanisms underlying ADHF-related renal injury. Additionally, in a cisplatin-induced acute kidney injury mouse model, increased ANGPTL4 expression was observed alongside reduced LPL activity, predominantly in the proximal tubular segments (Li et al., 2012), suggesting that ANGPTL4 may be involved in the AKI mechanism by regulating LPL activity. However, definitive mechanistic studies and clinical validation are currently lacking and warrant further exploration.

Analysis of plasma and urinary samples from patients with IgA nephropathy (IgAN) revealed significantly elevated levels of ANGPTL4, which was positively correlated with the extent of podocyte damage (Jia et al., 2020). These findings suggest the potential of ANGPTL4 as a tool for assessing the severity of IgAN and suggest its potential as a future therapeutic target for IgAN. Despite its importance, further large-scale studies are necessary to validate its clinical applicability.

Previous reports indicate that serum ANGPTL4 levels in patients undergoing chronic hemodialysis are more than five times higher than those in healthy controls (Baranowski et al., 2011), suggesting a significant role of ANGPTL4 in the dialysis process. Mahmood et al. reported a similar phenomenon: during chronic hemodialysis, the use of low-molecular-weight heparin for anticoagulation resulted in the release of ANGPTL4 from tissues into the bloodstream, whereas citric acid anticoagulation significantly reduced ANGPTL4 levels (Mahmood et al., 2014). These findings suggest that different anticoagulation strategies may differentially affect ANGPTL4 release and expression, although the exact mechanisms remain to be elucidated in future studies.

Our previous research preliminarily indicated that in an adenine-induced CKD interstitial fibrosis rat model, ANGPTL4 expression in renal tissue was significantly upregulated and positively correlated with renal injury markers (Li et al., 2023b). These findings suggest a potentially crucial role for ANGPTL4 in the pathology of CKD-related interstitial fibrosis. Subsequent studies in hypoxia-induced human renal tubular epithelial cell fibrosis revealed that both knockdown and overexpression of ANGPTL4 could either alleviate or exacerbate cell fibrosis progression, involving a regulatory loop with HIF-1α in CKD interstitial fibrosis progression (Li et al., 2024). These findings indicate that ANGPTL4 may be a key factor in CKD interstitial fibrosis. However, further *in vivo* and *in vitro* studies are needed to elucidate its precise mechanisms and assess its potential as a diagnostic and therapeutic target for renal fibrosis.

## 4 Other ANGPTLs in renal diseases

In addition to ANGPTL4, other members of the ANGPTL family play critical roles in renal diseases. Current research on ANGPTL3 has made significant strides, particularly in DKD and nephrotic syndrome. Studies by Ma et al. demonstrated that the deletion of ANGPTL3 ameliorates podocyte injury, epithelial-to-mesenchymal transition, and macrophage polarization from the M1 phenotype to the M2 phenotype by modulating the NLRP3 signaling pathway, revealing a novel immune mechanism underlying diabetic kidney damage (Ma et al., 2023b). Moreover, adjunctive therapy with anti-ANGPTL3 antibodies or the absence of ANGPTL3 significantly alleviates podocyte injury in DKD mouse models (Ma Q. et al., 2022; Ma et al., 2023c). In non-DKD individuals, ANGPTL3 deletion or suppression similarly has protective effects. In models of nephropathy induced by adriamycin or puromycin, as well as LPS-induced AKI, the inhibition of ANGPTL3 markedly reduces proteinuria, podocyte apoptosis, and renal functional impairment (Zhao et al., 2021; Ma Y. et al., 2022; Ji et al., 2023). Furthermore, downregulation of ANGPTL3 activates the PI3K/AKT signaling pathway, inhibiting TGF-β1-induced renal interstitial fibrosis (Yang et al., 2024). In addition, ANGPTL2 deficiency or knockdown has been shown to have protective effects on unilateral ureteral obstruction and hypoxia/reoxygenation-induced injury models (Morinaga et al., 2016; Xiang et al., 2020). ANGPTL2 also plays a role in immune modulation, with its deficiency promoting CD8^+^ T-cell infiltration and delaying tumor progression in RCC models (Kadomatsu et al., 2023). ANGPTL8, which is predominantly associated with metabolic kidney diseases, is correlated with an increased risk of renal dysfunction, particularly DKD, where it holds potential as a prognostic biomarker (Issa et al., 2019; Meng et al., 2021).

In summary, other members of the ANGPTL family, including ANGPTL2, ANGPTL3, and ANGPTL8, also contribute to the regulation of inflammation, fibrosis, and metabolic disturbances in kidney diseases. Future research should aim to elucidate their precise molecular mechanisms and develop targeted interventions for specific ANGPTL family members, thereby advancing therapeutic strategies for renal disorders.

## 5 Conclusion and perspectives

Current evidence indicates that ANGPTL4 plays a crucial role in the pathogenesis and progression of various kidney diseases. It may contribute to renal pathology through multiple mechanisms, including the regulation of inflammation, apoptosis, oxidative stress, hypoxia, tumor growth, and angiogenesis. Elevated ANGPTL4 expression has been observed in renal tissues across various kidney diseases, and its inhibition has shown promise in effectively halting disease progression in both *in vitro* and *in vivo* models, making ANGPTL4 an appealing therapeutic target. However, the precise mechanisms through which ANGPTL4 affects different kidney diseases remain inadequately understood. Increasing data suggest that ANGPTL4 levels in blood and/or urine could serve as biomarkers for multiple renal disorders, although issues such as sample size, individual variability, and reagent differences present ongoing debates. Future research with larger cohorts is essential for confirming its role in the early dynamic detection of kidney diseases. Additionally, targeting ANGPTL4 with approaches such as recombinant mutant human angiopoietin-like 4, anti-ANGPTL4 antibodies, and sialic acid precursor therapy could offer novel treatment strategies, although their safety and efficacy require further investigation.

## References

[B1] Adachi H. Fujiwara Y. Kondo T. Nishikawa T. Ogawa R. Matsumura T. (2009). Angptl 4 deficiency improves lipid metabolism, suppresses foam cell formation and protects against atherosclerosis. Biochem. Biophys. Res. Commun. 379 (4), 806–811. 10.1016/j.bbrc.2008.12.018 19094966

[B2] Alex S. Lange K. Amolo T. Grinstead J. S. Haakonsson A. K. Szalowska E. (2013). Short-chain fatty acids stimulate angiopoietin-like 4 synthesis in human colon adenocarcinoma cells by activating peroxisome proliferator-activated receptor γ. Mol. Cell. Biol. 33 (7), 1303–1316. 10.1128/mcb.00858-12 23339868 PMC3624264

[B3] Al Shawaf E. Abu-Farha M. Devarajan S. Alsairafi Z. Al-Khairi I. Cherian P. (2019). ANGPTL4: a predictive marker for diabetic nephropathy. J. Diabetes Res. 2019, 4943191. 10.1155/2019/4943191 31772941 PMC6854918

[B4] Anders H. J. Saxena R. Zhao M. H. Parodis I. Salmon J. E. Mohan C. (2020). Lupus nephritis. Nat. Rev. Dis. Prim. 6 (1), 7. 10.1038/s41572-019-0141-9 31974366

[B5] Apanovich N. Apanovich P. Mansorunov D. Kuzevanova A. Matveev V. Karpukhin A. (2021). The choice of candidates in survival markers based on coordinated gene expression in renal cancer. Front. Oncol. 11, 615787. 10.3389/fonc.2021.615787 34046336 PMC8144703

[B6] Apanovich N. Peters M. Apanovich P. Mansorunov D. Markova A. Matveev V. (2020). The genes-candidates for prognostic markers of metastasis by expression level in clear cell renal cell cancer. Diagn. (Basel) 10 (1), 30. 10.3390/diagnostics10010030 PMC7168144 31936274

[B7] Aryal B. Singh A. K. Zhang X. Varela L. Rotllan N. Goedeke L. (2018). Absence of ANGPTL4 in adipose tissue improves glucose tolerance and attenuates atherogenesis. JCI Insight 3 (6), e97918. 10.1172/jci.insight.97918 29563332 PMC5926923

[B8] Avalle L. Raggi L. Monteleone E. Savino A. Viavattene D. Statello L. (2022). STAT3 induces breast cancer growth via ANGPTL4, MMP13 and STC1 secretion by cancer associated fibroblasts. Oncogene 41 (10), 1456–1467. 10.1038/s41388-021-02172-y 35042959

[B9] Baba K. Kitajima Y. Miyake S. Nakamura J. Wakiyama K. Sato H. (2017). Hypoxia-induced ANGPTL4 sustains tumour growth and anoikis resistance through different mechanisms in scirrhous gastric cancer cell lines. Sci. Rep. 7 (1), 11127. 10.1038/s41598-017-11769-x 28894280 PMC5594024

[B10] Bano G. Imam M. T. Bajpai R. Alem G. Kashyap V. K. Habib A. (2023). Expression of angiopoetin-like protein-4 and kidney injury molecule-1 as preliminary diagnostic markers for diabetes-related kidney disease: a single center-based cross-sectional study. J. Pers. Med. 13 (4), 577. 10.3390/jpm13040577 37108963 PMC10146969

[B11] Baranowski T. Kralisch S. Bachmann A. Lössner U. Kratzsch J. Blüher M. (2011). Serum levels of the adipokine fasting-induced adipose factor/angiopoietin-like protein 4 depend on renal function. Horm. Metab. Res. 43 (2), 117–120. 10.1055/s-0030-1267917 20972945

[B12] Bose M. Almas S. Prabhakar S. (2017). Wnt signaling and podocyte dysfunction in diabetic nephropathy. J. Investig. Med. 65 (8), 1093–1101. 10.1136/jim-2017-000456 28935636

[B13] Cara-Fuentes G. Segarra A. Silva-Sanchez C. Wang H. Lanaspa M. A. Johnson R. J. (2017). Angiopoietin-like-4 and minimal change disease. PLoS One 12 (4), e0176198. 10.1371/journal.pone.0176198 28441404 PMC5404758

[B14] Chaube B. Citrin K. M. Sahraei M. Singh A. K. de Urturi D. S. Ding W. (2023). Suppression of angiopoietin-like 4 reprograms endothelial cell metabolism and inhibits angiogenesis. Nat. Commun. 14 (1), 8251. 10.1038/s41467-023-43900-0 38086791 PMC10716292

[B15] Chen J. S. Xie P. F. Feng H. (2023). The role of exercise in improving hyperlipidemia-renal injuries induced by a high-fat diet: a literature review. PeerJ 11, e15435. 10.7717/peerj.15435 37283893 PMC10239619

[B16] Chen T. C. Benjamin D. I. Kuo T. Lee R. A. Li M. L. Mar D. J. (2017). The glucocorticoid-Angptl4-ceramide axis induces insulin resistance through PP2A and PKCζ. Sci. Signal 10 (489), eaai7905. 10.1126/scisignal.aai7905 28743803 PMC6218395

[B17] Cheng H. S. Yip Y. S. Lim E. K. Y. Wahli W. Tan N. S. (2021). PPARs and tumor microenvironment: the emerging roles of the metabolic master regulators in tumor stromal-epithelial crosstalk and carcinogenesis. Cancers (Basel) 13 (9), 2153. 10.3390/cancers13092153 33946986 PMC8125182

[B18] Cho D. I. Kang H. J. Jeon J. H. Eom G. H. Cho H. H. Kim M. R. (2019). Antiinflammatory activity of ANGPTL4 facilitates macrophage polarization to induce cardiac repair. JCI Insight 4 (16), e125437. 10.1172/jci.insight.125437 31434807 PMC6777833

[B19] Chong H. C. Chan J. S. Goh C. Q. Gounko N. V. Luo B. Wang X. (2014). Angiopoietin-like 4 stimulates STAT3-mediated iNOS expression and enhances angiogenesis to accelerate wound healing in diabetic mice. Mol. Ther. 22 (9), 1593–1604. 10.1038/mt.2014.102 24903577 PMC4435481

[B20] Chugh S. S. Clement L. C. (2023). Idiopathic minimal change nephrotic syndrome: a podocyte mystery nears the end. Am. J. Physiol. Ren. Physiol. 325 (6), F685–f694. 10.1152/ajprenal.00219.2023 PMC10878723 37795536

[B21] Chugh S. S. Clement L. C. Macé C. (2012). New insights into human minimal change disease: lessons from animal models. Am. J. Kidney Dis. 59 (2), 284–292. 10.1053/j.ajkd.2011.07.024 21974967 PMC3253318

[B22] Chugh S. S. Macé C. Clement L. C. Del Nogal Avila M. Marshall C. B. (2014). Angiopoietin-like 4 based therapeutics for proteinuria and kidney disease. Front. Pharmacol. 5, 23. 10.3389/fphar.2014.00023 24611049 PMC3933785

[B23] Clement L. C. Avila-Casado C. Macé C. Soria E. Bakker W. W. Kersten S. (2011). Podocyte-secreted angiopoietin-like-4 mediates proteinuria in glucocorticoid-sensitive nephrotic syndrome. Nat. Med. 17 (1), 117–122. 10.1038/nm.2261 21151138 PMC3021185

[B24] Clement L. C. Macé C. Avila-Casado C. Joles J. A. Kersten S. Chugh S. S. (2014). Circulating angiopoietin-like 4 links proteinuria with hypertriglyceridemia in nephrotic syndrome. Nat. Med. 20 (1), 37–46. 10.1038/nm.3396 24317117 PMC4114723

[B25] Clement L. C. Macé C. Del Nogal Avila M. Marshall C. B. Chugh S. S. (2015). The proteinuria-hypertriglyceridemia connection as a basis for novel therapeutics for nephrotic syndrome. Transl. Res. 165 (4), 499–504. 10.1016/j.trsl.2014.06.004 25005737 PMC4270958

[B26] Cohen H. T. McGovern F. J. (2005). Renal-cell carcinoma. N. Engl. J. Med. 353 (23), 2477–2490. 10.1056/NEJMra043172 16339096

[B27] Del Nogal-Avila M. Donoro-Blazquez H. Saha M. K. Marshall C. B. Clement L. C. Macé C. E. (2016). Novel therapeutic approaches for chronic kidney disease due to glomerular disorders. Am. J. Physiol. Ren. Physiol. 311 (1), F63–F65. 10.1152/ajprenal.00245.2016 PMC4967169 27147672

[B28] Desai U. Lee E. C. Chung K. Gao C. Gay J. Key B. (2007). Lipid-lowering effects of anti-angiopoietin-like 4 antibody recapitulate the lipid phenotype found in angiopoietin-like 4 knockout mice. Proc. Natl. Acad. Sci. U. S. A. 104 (28), 11766–11771. 10.1073/pnas.0705041104 17609370 PMC1913890

[B29] Dewey F. E. Gusarova V. O'Dushlaine C. Gottesman O. Trejos J. Hunt C. (2016). Inactivating variants in ANGPTL4 and risk of coronary artery disease. N. Engl. J. Med. 374 (12), 1123–1133. 10.1056/NEJMoa1510926 26933753 PMC4900689

[B30] Dong D. Jia L. Zhou Y. Ren L. Li J. Zhang J. (2017). Serum level of ANGPTL4 as a potential biomarker in renal cell carcinoma. Urol. Oncol. 35 (5), 279–285. 10.1016/j.urolonc.2016.12.017 28110976

[B31] Drager L. F. Yao Q. Hernandez K. L. Shin M. K. Bevans-Fonti S. Gay J. (2013). Chronic intermittent hypoxia induces atherosclerosis via activation of adipose angiopoietin-like 4. Am. J. Respir. Crit. Care Med. 188 (2), 240–248. 10.1164/rccm.201209-1688OC 23328524 PMC3778753

[B32] Fernández-Hernando C. Suárez Y. (2020). ANGPTL4: a multifunctional protein involved in metabolism and vascular homeostasis. Curr. Opin. Hematol. 27 (3), 206–213. 10.1097/moh.0000000000000580 32205586 PMC9013473

[B33] Gao R. Zhou P. Li Y. Li Q. (2023). High glucose-induced IL-7/IL-7R upregulation of dermal fibroblasts inhibits angiogenesis in a paracrine way in delayed diabetic wound healing. J. Cell. Commun. Signal 17 (3), 1023–1038. 10.1007/s12079-023-00754-x 37217704 PMC10409704

[B34] Gao X. Zhang M. Feng W. Xu Z. Wang Y. Shi L. (2020). Alteration of angiopoietin-like protein 4 levels in serum or urine correlate with different biochemical markers in hyperlipidemia-related proteinuria. Biomed. Res. Int. 2020, 5281251. 10.1155/2020/5281251 32280690 PMC7125447

[B35] Gao Y. Ma Y. Xie D. Jiang H. (2022). ManNAc protects against podocyte pyroptosis via inhibiting mitochondrial damage and ROS/NLRP3 signaling pathway in diabetic kidney injury model. Int. Immunopharmacol. 107, 108711. 10.1016/j.intimp.2022.108711 35338958

[B36] Ge H. Yang G. Huang L. Motola D. L. Pourbahrami T. Li C. (2004). Oligomerization and regulated proteolytic processing of angiopoietin-like protein 4. J. Biol. Chem. 279 (3), 2038–2045. 10.1074/jbc.M307583200 14570927

[B37] Goh Y. Y. Pal M. Chong H. C. Zhu P. Tan M. J. Punugu L. (2010). Angiopoietin-like 4 interacts with integrins beta1 and beta5 to modulate keratinocyte migration. Am. J. Pathol. 177 (6), 2791–2803. 10.2353/ajpath.2010.100129 20952587 PMC2993291

[B38] Gomez Perdiguero E. Liabotis-Fontugne A. Durand M. Faye C. Ricard-Blum S. Simonutti M. (2016). ANGPTL4-αvβ3 interaction counteracts hypoxia-induced vascular permeability by modulating Src signalling downstream of vascular endothelial growth factor receptor 2. J. Pathol. 240 (4), 461–471. 10.1002/path.4805 27577973

[B39] González-Muniesa P. de Oliveira C. Pérez de Heredia F. Thompson M. P. Trayhurn P. (2011). Fatty acids and hypoxia stimulate the expression and secretion of the adipokine ANGPTL4 (angiopoietin-like protein 4/fasting-induced adipose factor) by human adipocytes. J. Nutr. Nutr. 4 (3), 146–153. 10.1159/000327774 21709421

[B40] Grootaert C. Van de Wiele T. Verstraete W. Bracke M. Vanhoecke B. (2012). Angiopoietin-like protein 4: health effects, modulating agents and structure-function relationships. Expert Rev. Proteomics 9 (2), 181–199. 10.1586/epr.12.12 22462789

[B41] Guo K. Pan P. Wu M. Ma Y. Lu J. Chen H. (2020). Hyposialylated angiopoietin-like-4 induces apoptosis of podocytes via β1 Integrin/FAK signaling in diabetic nephropathy. Mol. Cell. Endocrinol. 505, 110730. 10.1016/j.mce.2020.110730 31981598

[B42] Hamilton M. J. Young M. Jang K. Sauer S. Neang V. E. King A. T. (2020). HOTAIRM1 lncRNA is downregulated in clear cell renal cell carcinoma and inhibits the hypoxia pathway. Cancer Lett. 472, 50–58. 10.1016/j.canlet.2019.12.022 31862408 PMC6992348

[B43] Han X. Song D. (2022). Using a machine learning approach to identify key biomarkers for renal clear cell carcinoma. Int. J. Gen. Med. 15, 3541–3558. 10.2147/ijgm.S351168 35392028 PMC8980298

[B44] Hato T. Tabata M. Oike Y. (2008). The role of angiopoietin-like proteins in angiogenesis and metabolism. Trends Cardiovasc Med. 18 (1), 6–14. 10.1016/j.tcm.2007.10.003 18206803

[B45] Hjelholt A. J. Søndergaard E. Pedersen S. B. Møller N. Jessen N. Jørgensen J. O. L. (2020). Growth hormone upregulates ANGPTL4 mRNA and suppresses lipoprotein lipase via fatty acids: randomized experiments in human individuals. Metabolism 105, 154188. 10.1016/j.metabol.2020.154188 32084431

[B46] Hsieh J. J. Purdue M. P. Signoretti S. Swanton C. Albiges L. Schmidinger M. (2017). Renal cell carcinoma. Nat. Rev. Dis. Prim. 3, 17009. 10.1038/nrdp.2017.9 28276433 PMC5936048

[B47] Huang R. L. Teo Z. Chong H. C. Zhu P. Tan M. J. Tan C. K. (2011). ANGPTL4 modulates vascular junction integrity by integrin signaling and disruption of intercellular VE-cadherin and claudin-5 clusters. Blood 118 (14), 3990–4002. 10.1182/blood-2011-01-328716 21841165

[B48] Issa Y. A. Abd ElHafeez S. S. Amin N. G. (2019). The potential role of angiopoietin-like protein-8 in type 2 diabetes mellitus: a possibility for predictive diagnosis and targeted preventive measures? Epma J. 10 (3), 239–248. 10.1007/s13167-019-00180-3 31462941 PMC6695457

[B49] Ji B. Liu J. Ma Y. Yin Y. Xu H. Shen Q. (2023). Minnelide combined with Angptl3 knockout completely protects mice with adriamycin nephropathy via suppression of TGF-β1-Smad2 and p53 pathways. Int. Immunopharmacol. 115, 109656. 10.1016/j.intimp.2022.109656 36608441

[B50] Jia S. Peng X. Liang L. Zhang Y. Li M. Zhou Q. (2020). The study of angptl4-modulated podocyte injury in IgA nephropathy. Front. Physiol. 11, 575722. 10.3389/fphys.2020.575722 33643055 PMC7905042

[B51] Jiang Q. Miao R. Wang Y. Wang W. Zhao D. Niu Y. (2023). ANGPTL4 inhibits granulosa cell proliferation in polycystic ovary syndrome by EGFR/JAK1/STAT3-mediated induction of p21. Faseb J. 37 (2), e22693. 10.1096/fj.202201246RR 36607250

[B52] Kadomatsu T. Hara C. Kurahashi R. Horiguchi H. Morinaga J. Miyata K. (2023). ANGPTL2-mediated epigenetic repression of MHC-I in tumor cells accelerates tumor immune evasion. Mol. Oncol. 17 (12), 2637–2658. 10.1002/1878-0261.13490 37452654 PMC10701769

[B53] Kang Y. T. Hsu W. C. Ou C. C. Tai H. C. Hsu H. T. Yeh K. T. (2020). Metformin mitigates nickel-elicited angiopoietin-like protein 4 expression via HIF-1α for lung tumorigenesis. Int. J. Mol. Sci. 21 (2), 619. 10.3390/ijms21020619 31963541 PMC7014330

[B54] Kang Y. T. Li C. T. Tang S. C. Hsin I. L. Lai Y. C. Hsiao Y. P. (2021). Nickel chloride regulates ANGPTL4 via the HIF-1α-mediated TET1 expression in lung cells. Toxicol. Lett. 352, 17–25. 10.1016/j.toxlet.2021.09.007 34571076

[B55] Katanasaka Y. Kodera Y. Kitamura Y. Morimoto T. Tamura T. Koizumi F. (2013). Epidermal growth factor receptor variant type III markedly accelerates angiogenesis and tumor growth via inducing c-myc mediated angiopoietin-like 4 expression in malignant glioma. Mol. Cancer 12, 31. 10.1186/1476-4598-12-31 23617883 PMC3641008

[B56] Kersten S. (2021). Role and mechanism of the action of angiopoietin-like protein ANGPTL4 in plasma lipid metabolism. J. Lipid Res. 62, 100150. 10.1016/j.jlr.2021.100150 34801488 PMC8666355

[B57] Kersten S. Mandard S. Tan N. S. Escher P. Metzger D. Chambon P. (2000). Characterization of the fasting-induced adipose factor FIAF, a novel peroxisome proliferator-activated receptor target gene. J. Biol. Chem. 275 (37), 28488–28493. 10.1074/jbc.M004029200 10862772

[B58] Kim I. Kim H. G. Kim H. Kim H. H. Park S. K. Uhm C. S. (2000). Hepatic expression, synthesis and secretion of a novel fibrinogen/angiopoietin-related protein that prevents endothelial-cell apoptosis. Biochem. J. 346 (Pt 3)**,** 603–610. 10.1042/0264-6021:3460603 10698685 PMC1220891

[B59] Kirsch N. Chang L. S. Koch S. Glinka A. Dolde C. Colozza G. (2017). Angiopoietin-like 4 is a Wnt signaling antagonist that promotes LRP6 turnover. Dev. Cell. 43 (1), 71–82. 10.1016/j.devcel.2017.09.011 29017031

[B60] Koliwad S. K. Kuo T. Shipp L. E. Gray N. E. Backhed F. So A. Y. (2009). Angiopoietin-like 4 (ANGPTL4, fasting-induced adipose factor) is a direct glucocorticoid receptor target and participates in glucocorticoid-regulated triglyceride metabolism. J. Biol. Chem. 284 (38), 25593–25601. 10.1074/jbc.M109.025452 19628874 PMC2757961

[B61] Le Jan S. Amy C. Cazes A. Monnot C. Lamandé N. Favier J. (2003). Angiopoietin-like 4 is a proangiogenic factor produced during ischemia and in conventional renal cell carcinoma. Am. J. Pathol. 162 (5), 1521–1528. 10.1016/s0002-9440(10)64285-x 12707035 PMC1851201

[B62] Li L. Chao Z. Waikeong U. Xiao J. Ge Y. Wang Y. (2023a). Metabolic classifications of renal cell carcinoma reveal intrinsic connections with clinical and immune characteristics. J. Transl. Med. 21 (1), 146. 10.1186/s12967-023-03978-y 36829161 PMC9960222

[B63] Li L. Chong H. C. Ng S. Y. Kwok K. W. Teo Z. Tan E. H. P. (2015). Angiopoietin-like 4 increases pulmonary tissue leakiness and damage during influenza pneumonia. Cell. Rep. 10 (5), 654–663. 10.1016/j.celrep.2015.01.011 25660016 PMC7185373

[B64] Li S. Nagothu K. Ranganathan G. Ali S. M. Shank B. Gokden N. (2012). Reduced kidney lipoprotein lipase and renal tubule triglyceride accumulation in cisplatin-mediated acute kidney injury. Am. J. Physiol. Ren. Physiol. 303 (3), F437–F448. 10.1152/ajprenal.00111.2012 PMC3433869 22622461

[B65] Li Y. Chen S. Yang Q. Liu X. Zhou W. Kang T. (2024). The ANGPTL4-HIF-1α loop: a critical regulator of renal interstitial fibrosis. J. Transl. Med. 22 (1), 649. 10.1186/s12967-024-05466-3 38992710 PMC11241841

[B66] Li Y. Gong W. Liu J. Chen X. Suo Y. Yang H. (2022). Angiopoietin-like protein 4 promotes hyperlipidemia-induced renal injury by down-regulating the expression of ACTN4. Biochem. Biophys. Res. Commun. 595, 69–75. 10.1016/j.bbrc.2022.01.061 35101665

[B67] Li Y. Mao H. Kang T. Zhang L. Liu Q. Ou S. (2023b). Angiopoietin-like protein 4 signaling pathway's expression and functionality in the adenine-induced chronic renal disease rat model. Chin. J. Neahrol (02), 126–134. 10.3760/cma.j.cn441217-20220708-00712

[B68] Li Y. Xu Z. Deng H. Liu M. Lin X. Zhang M. (2023c). ANGPTL4 promotes nephrotic syndrome by downregulating podocyte expression of ACTN4 and podocin. Biochem. Biophys. Res. Commun. 639, 176–182. 10.1016/j.bbrc.2022.11.081 36495766

[B69] Liabotis A. Ardidie-Robouant C. Mailly P. Besbes S. Gutierrez C. Atlas Y. (2022). Angiopoietin-like 4-induced 3D capillary morphogenesis correlates to stabilization of endothelial adherens junctions and restriction of VEGF-induced sprouting. Biomedicines 10 (2), 206. 10.3390/biomedicines10020206 35203415 PMC8869696

[B70] Liu G. He L. (2019). Epigallocatechin-3-Gallate attenuates adriamycin-induced focal segmental glomerulosclerosis via suppression of oxidant stress and apoptosis by targeting hypoxia-inducible factor-1α/angiopoietin-like 4 pathway. Pharmacology 103 (5-6), 303–314. 10.1159/000496799 30840953

[B71] Liu Y. (2011). Cellular and molecular mechanisms of renal fibrosis. Nat. Rev. Nephrol. 7 (12), 684–696. 10.1038/nrneph.2011.149 22009250 PMC4520424

[B72] Liu Y. Hamid N. Manzoor R. Zhang B. F. Liao Y. L. Wang J. X. (2023). PPARβ/δ-ANGPTL4 axis mediates the promotion of mono-2-ethylhexyl phthalic acid on MYCN-amplified neuroblastoma development. Sci. Total Environ. 912, 168949. 10.1016/j.scitotenv.2023.168949 38042186

[B73] Ljungberg B. (2007). Prognostic markers in renal cell carcinoma. Curr. Opin. Urol. 17 (5), 303–308. 10.1097/MOU.0b013e328277f180 17762621

[B74] Lu B. Moser A. Shigenaga J. K. Grunfeld C. Feingold K. R. (2010). The acute phase response stimulates the expression of angiopoietin like protein 4. Biochem. Biophys. Res. Commun. 391 (4), 1737–1741. 10.1016/j.bbrc.2009.12.145 20043872

[B75] Lu R. Zhou J. Liu B. Liang N. He Y. Bai L. (2017). Paeoniflorin ameliorates Adriamycin-induced nephrotic syndrome through the PPARγ/ANGPTL4 pathway *in vivo* and vitro. Biomed. Pharmacother. 96, 137–147. 10.1016/j.biopha.2017.09.105 28972886

[B76] Luo D. Li J. Hu M. Wang Y. Pi P. Ning M. (2023). Angiopoietin-like 4 (ANGPTL4) suppression ameliorates lupus nephritis in MRL/lpr mice by inactivating NLRP3 inflammasome and inhibiting inflammatory response. Iran. J. Immunol. 3 (20), 316–326. 10.22034/iji.2023.97942.2541 37543838

[B77] Lv J. C. Zhang L. X. (2019). Prevalence and disease burden of chronic kidney disease. Adv. Exp. Med. Biol. 1165, 3–15. 10.1007/978-981-13-8871-2_1 31399958

[B78] Ma B. Qin L. Sun Z. Wang J. Tran L. J. Zhang J. (2023a). The single-cell evolution trajectory presented different hypoxia heterogeneity to reveal the carcinogenesis of genes in clear cell renal cell carcinoma: based on multiple omics and real experimental verification. Environ. Toxicol. 39, 869–881. 10.1002/tox.24009 37886854

[B79] Ma J. Chen X. Li J. S. Peng L. Wei S. Y. Zhao S. L. (2015). Upregulation of podocyte-secreted angiopoietin-like-4 in diabetic nephropathy. Endocrine 49 (2), 373–384. 10.1007/s12020-014-0486-5 25424436

[B80] Ma Q. Hu X. Liu F. Cao Z. Han L. Zhou K. (2022a). A novel fusion protein consisting of anti-ANGPTL3 antibody and interleukin-22 ameliorates diabetic nephropathy in mice. Front. Immunol. 13, 1011442. 10.3389/fimmu.2022.1011442 36544775 PMC9760875

[B81] Ma Y. Chen Y. Xu H. Du N. (2023b). The influence of angiopoietin-like protein 3 on macrophages polarization and its effect on the podocyte EMT in diabetic nephropathy. Front. Immunol. 14, 1228399. 10.3389/fimmu.2023.1228399 37638046 PMC10450617

[B82] Ma Y. Liu J. Liu H. Han X. Sun L. Xu H. (2022b). Podocyte protection by Angptl3 knockout via inhibiting ROS/GRP78 pathway in LPS-induced acute kidney injury. Int. Immunopharmacol. 105, 108549. 10.1016/j.intimp.2022.108549 35086056

[B83] Ma Y. Xie D. Liu J. Han X. Xu H. Chen Y. (2023c). Angiopoietin-like protein 3 deficiency combined with valsartan administration protects better against podocyte damage in streptozotocin-induced diabetic nephropathy mice. Int. Immunopharmacol. 115, 109715. 10.1016/j.intimp.2023.109715 37724955

[B84] Macé C. Chugh S. S. (2014). Nephrotic syndrome: components, connections, and angiopoietin-like 4-related therapeutics. J. Am. Soc. Nephrol. 25 (11), 2393–2398. 10.1681/asn.2014030267 24854282 PMC4214538

[B85] Macé C. Del Nogal Avila M. Marshall C. B. Kharlyngdoh J. Das R. Molina-Jijon E. (2020). The zinc fingers and homeoboxes 2 protein ZHX2 and its interacting proteins regulate upstream pathways in podocyte diseases. Kidney Int. 97 (4), 753–764. 10.1016/j.kint.2019.11.011 32059999 PMC7394365

[B86] Mahmood D. Makoveichuk E. Nilsson S. Olivecrona G. Stegmayr B. (2014). Response of angiopoietin-like proteins 3 and 4 to hemodialysis. Int. J. Artif. Organs 37 (1), 13–20. 10.5301/ijao.5000252 24634330

[B87] Makoveichuk E. Vorrsjö E. Olivecrona T. Olivecrona G. (2017). TNF-α decreases lipoprotein lipase activity in 3T3-L1 adipocytes by up-regulation of angiopoietin-like protein 4. Biochim. Biophys. Acta Mol. Cell. Biol. Lipids 1862 (5), 533–540. 10.1016/j.bbalip.2017.02.005 28215713

[B88] Mandard S. Zandbergen F. van Straten E. Wahli W. Kuipers F. Müller M. (2006). The fasting-induced adipose factor/angiopoietin-like protein 4 is physically associated with lipoproteins and governs plasma lipid levels and adiposity. J. Biol. Chem. 281 (2), 934–944. 10.1074/jbc.M506519200 16272564

[B89] Meng X. Zou H. Li D. Yu P. Huang L. Zhang J. (2021). Association of circulating ANGPTL8 levels with renal dysfunction: a case-control study. Front. Public Health 9, 710504. 10.3389/fpubh.2021.710504 34557469 PMC8452901

[B90] Mi X. Ning Y. Wang X. Junjvlieke Z. Wang L. Wang S. (2019). GR and Foxa1 promote the transcription of ANGPTL4 in bovine adipocytes. Mol. Cell. Probes 48, 101443. 10.1016/j.mcp.2019.101443 31487539

[B91] Mizuno S. Seishima R. Yamasaki J. Hattori K. Ogiri M. Matsui S. (2022). Angiopoietin-like 4 promotes glucose metabolism by regulating glucose transporter expression in colorectal cancer. J. Cancer Res. Clin. Oncol. 148 (6), 1351–1361. 10.1007/s00432-022-03960-z 35195748 PMC11800850

[B92] Moorhead J. F. Chan M. K. El-Nahas M. Varghese Z. (1982). Lipid nephrotoxicity in chronic progressive glomerular and tubulo-interstitial disease. Lancet 2 (8311), 1309–1311. 10.1016/s0140-6736(82)91513-6 6128601

[B93] Morinaga J. Kadomatsu T. Miyata K. Endo M. Terada K. Tian Z. (2016). Angiopoietin-like protein 2 increases renal fibrosis by accelerating transforming growth factor-β signaling in chronic kidney disease. Kidney Int. 89 (2), 327–341. 10.1016/j.kint.2015.12.021 26806834

[B94] National Library of Medicine (2002). K/DOQI clinical practice guidelines for chronic kidney disease: evaluation, classification, and stratification. Am. J. Kidney Dis. 39 (2 Suppl. 1), S1–S266. 10.1053/ajkd.2002.30939 11904577

[B95] Niu Y. Bao L. Chen Y. Wang C. Luo M. Zhang B. (2020). HIF2-Induced long noncoding RNA RAB11B-AS1 promotes hypoxia-mediated angiogenesis and breast cancer metastasis. Cancer Res. 80 (5), 964–975. 10.1158/0008-5472.Can-19-1532 31900259 PMC7056556

[B96] Nyrén R. Makoveichuk E. Malla S. Kersten S. Nilsson S. K. Ericsson M. (2019). Lipoprotein lipase in mouse kidney: effects of nutritional status and high-fat diet. Am. J. Physiol. Ren. Physiol. 316 (3), F558–f571. 10.1152/ajprenal.00474.2018 30698048

[B97] Ogurtsova K. da Rocha Fernandes J. D. Huang Y. Linnenkamp U. Guariguata L. Cho N. H. (2017). IDF Diabetes Atlas: global estimates for the prevalence of diabetes for 2015 and 2040. Diabetes Res. Clin. Pract. 128, 40–50. 10.1016/j.diabres.2017.03.024 28437734

[B98] Okamoto H. Cavino K. Na E. Krumm E. Kim S. Stevis P. E. (2017). Angptl4 does not control hyperglucagonemia or α-cell hyperplasia following glucagon receptor inhibition. Proc. Natl. Acad. Sci. U. S. A. 114 (10), 2747–2752. 10.1073/pnas.1620989114 28143927 PMC5347591

[B99] Okochi-Takada E. Hattori N. Tsukamoto T. Miyamoto K. Ando T. Ito S. (2014). ANGPTL4 is a secreted tumor suppressor that inhibits angiogenesis. Oncogene 33 (17), 2273–2278. 10.1038/onc.2013.174 23686315

[B100] Pan D. S. Wang W. Liu N. S. Yang Q. J. Zhang K. Zhu J. Z. (2017). Chiglitazar preferentially regulates gene expression via configuration-restricted binding and phosphorylation inhibition of PPARγ. PPAR Res. 2017, 4313561. 10.1155/2017/4313561 29056962 PMC5625810

[B101] Phua T. Sng M. K. Tan E. H. Chee D. S. Li Y. Wee J. W. (2017). Angiopoietin-like 4 mediates colonic inflammation by regulating chemokine transcript stability via tristetraprolin. Sci. Rep. 7, 44351. 10.1038/srep44351 28287161 PMC5347094

[B102] Qi X. Bie M. Jiang R. Kang F. (2023). HIF-1α regulates osteoclastogenesis and alveolar bone resorption in periodontitis via ANGPTL4. Arch. Oral Biol. 153, 105736. 10.1016/j.archoralbio.2023.105736 37290266

[B103] Qin L. Zhang R. Yang S. Chen F. Shi J. (2019). Knockdown of ANGPTL-4 inhibits inflammatory response and extracellular matrix accumulation in glomerular mesangial cells cultured under high glucose condition. Artif. Cells Nanomed Biotechnol. 47 (1), 3368–3373. 10.1080/21691401.2019.1649274 31387395

[B104] Qiu W. Huang L. Li Y. Liu Q. Lv Y. (2023). Dysregulation of angiopoietin-like-4 associated with hyperlipidemia-induced renal injury by AMPK/ACC pathway. Curr. Pharm. Des. 29 (4), 300–309. 10.2174/1381612829666221219123937 36537603

[B105] Qiu Z. Yang J. Deng G. Li D. Zhang S. (2021). Angiopoietin-like 4 promotes angiogenesis and neurogenesis in a mouse model of acute ischemic stroke. Brain Res. Bull. 168, 156–164. 10.1016/j.brainresbull.2020.12.023 33417949

[B106] Rademaker M. T. Pilbrow A. P. Ellmers L. J. Palmer S. C. Davidson T. Mbikou P. (2021). Acute decompensated heart failure and the kidney: physiological, histological and transcriptomic responses to development and recovery. J. Am. Heart Assoc. 10 (18), e021312. 10.1161/jaha.121.021312 34533033 PMC8649508

[B107] Remuzzi G. Ruggenenti P. Benigni A. (1997). Understanding the nature of renal disease progression. Kidney Int. 51 (1), 2–15. 10.1038/ki.1997.2 8995712

[B108] Ruppert P. M. M. Michielsen C. Hazebroek E. J. Pirayesh A. Olivecrona G. Afman L. A. (2020). Fasting induces ANGPTL4 and reduces LPL activity in human adipose tissue. Mol. Metab. 40, 101033. 10.1016/j.molmet.2020.101033 32504883 PMC7334813

[B109] Saeedi P. Petersohn I. Salpea P. Malanda B. Karuranga S. Unwin N. (2019). Global and regional diabetes prevalence estimates for 2019 and projections for 2030 and 2045: results from the International Diabetes Federation Diabetes Atlas, 9(th) edition. Diabetes Res. Clin. Pract. 157, 107843. 10.1016/j.diabres.2019.107843 31518657

[B110] Shen X. Zhang Y. Lin C. Weng C. Wang Y. Feng S. (2020). Calcineurin inhibitors ameliorate PAN-induced podocyte injury through the NFAT-Angptl4 pathway. J. Pathol. 252 (3), 227–238. 10.1002/path.5512 32686149

[B111] Singh A. K. Aryal B. Chaube B. Rotllan N. Varela L. Horvath T. L. (2018). Brown adipose tissue derived ANGPTL4 controls glucose and lipid metabolism and regulates thermogenesis. Mol. Metab. 11, 59–69. 10.1016/j.molmet.2018.03.011 29627378 PMC6001401

[B112] Sodhi A. Ma T. Menon D. Deshpande M. Jee K. Dinabandhu A. (2019). Angiopoietin-like 4 binds neuropilins and cooperates with VEGF to induce diabetic macular edema. J. Clin. Investig. 129 (11), 4593–4608. 10.1172/jci120879 31545295 PMC6819094

[B113] Soliman S. Mohan C. (2017). Lupus nephritis biomarkers. Clin. Immunol. 185, 10–20. 10.1016/j.clim.2016.08.001 27498110

[B114] Tanaka Y. (2022). Systemic lupus erythematosus. Best. Pract. Res. Clin. Rheumatol. 36 (4), 101814. 10.1016/j.berh.2022.101814 36702700

[B115] Tang J. J. Li G. X. Liu Z. G. Yi R. Yu D. Zhang Y. B. (2022). Danlou tablet improves chronic intermittent hypoxia-induced dyslipidemia and arteriosclerosis by HIF-1α-Angptl4 mRNA signaling pathway. Chin. J. Integr. Med. 28 (6), 509–517. 10.1007/s11655-020-3255-8 32623702

[B116] Teo Z. Chan J. S. K. Chong H. C. Sng M. K. Choo C. C. Phua G. Z. M. (2017). Angiopoietin-like 4 induces a β-catenin-mediated upregulation of ID3 in fibroblasts to reduce scar collagen expression. Sci. Rep. 7 (1), 6303. 10.1038/s41598-017-05869-x 28740178 PMC5524754

[B117] The Lancet (2018). Global, regional, and national disability-adjusted life-years (DALYs) for 359 diseases and injuries and healthy life expectancy (HALE) for 195 countries and territories, 1990-2017: a systematic analysis for the Global Burden of Disease Study 2017. Lancet 392 (10159), 1859–1922. 10.1016/s0140-6736(18)32335-3 30415748 PMC6252083

[B118] The Lancet (2020). Global, regional, and national burden of chronic kidney disease, 1990-2017: a systematic analysis for the Global Burden of Disease Study 2017. Lancet 395 (10225), 709–733. 10.1016/s0140-6736(20)30045-3 32061315 PMC7049905

[B119] Tian M. M. Wang Y. S. Xiao H. B. (2022). Dual roles of ANGPTL4 in multiple inflammatory responses in stomatitis mice. Mol. Biol. Rep. 49 (10), 9195–9204. 10.1007/s11033-022-07745-y 35819554

[B120] Tomiyama-Hanayama M. Rakugi H. Kohara M. Mima T. Adachi Y. Ohishi M. (2009). Effect of interleukin-6 receptor blockage on renal injury in apolipoprotein E-deficient mice. Am. J. Physiol. Ren. Physiol. 297 (3), F679–F684. 10.1152/ajprenal.90680.2008 19570877

[B121] Vanarsa K. Soomro S. Zhang T. Strachan B. Pedroza C. Nidhi M. (2020). Quantitative planar array screen of 1000 proteins uncovers novel urinary protein biomarkers of lupus nephritis. Ann. Rheum. Dis. 79 (10), 1349–1361. 10.1136/annrheumdis-2019-216312 32651195 PMC7839323

[B122] Verine J. Lehmann-Che J. Soliman H. Feugeas J. P. Vidal J. S. Mongiat-Artus P. (2010). Determination of angptl4 mRNA as a diagnostic marker of primary and metastatic clear cell renal-cell carcinoma. PLoS One 5 (4), e10421. 10.1371/journal.pone.0010421 20454689 PMC2861680

[B123] Wang X. Qi D. Fu F. Li X. Liu Y. Ji K. (2019). Therapeutic and antiproteinuric effects of salvianolic acid A in combined with low-dose prednisone in minimal change disease rats: involvement of PPARγ/Angptl4 and Nrf2/HO-1 pathways. Eur. J. Pharmacol. 858, 172342. 10.1016/j.ejphar.2019.04.023 31129156

[B124] Wang Y. Liu L. M. Wei L. Ye W. W. Meng X. Y. Chen F. (2016). Angiopoietin-like protein 4 improves glucose tolerance and insulin resistance but induces liver steatosis in high-fat-diet mice. Mol. Med. Rep. 14 (4), 3293–3300. 10.3892/mmr.2016.5637 27573470

[B125] Wang Y. Wang Y. Liu F. (2018). A 44-gene set constructed for predicting the prognosis of clear cell renal cell carcinoma. Int. J. Mol. Med. 42 (6), 3105–3114. 10.3892/ijmm.2018.3899 30272265 PMC6202093

[B126] Wang Z. Wu Q. Wang H. Gao Y. Nie K. Tang Y. (2022). Diosgenin protects against podocyte injury in early phase of diabetic nephropathy through regulating SIRT6. Phytomedicine 104, 154276. 10.1016/j.phymed.2022.154276 35728388

[B127] Wood C. G. (2007). Multimodal approaches in the management of locally advanced and metastatic renal cell carcinoma: combining surgery and systemic therapies to improve patient outcome. Clin. Cancer Res. 13 (2 Pt 2), 697s–702s. 10.1158/1078-0432.Ccr-06-2109 17255296

[B128] Xiang H. Xue W. Li Y. Zheng J. Ding C. Dou M. (2020). Knockdown of ANGPTL2 protects renal tubular epithelial cells against hypoxia/reoxygenation-induced injury via suppressing TLR4/NF-κB signaling pathway and activating Nrf2/HO-1 signaling pathway. Cell. Transpl. 29, 963689720946663. 10.1177/0963689720946663 PMC7784569 32993399

[B129] Xu A. Lam M. C. Chan K. W. Wang Y. Zhang J. Hoo R. L. (2005). Angiopoietin-like protein 4 decreases blood glucose and improves glucose tolerance but induces hyperlipidemia and hepatic steatosis in mice. Proc. Natl. Acad. Sci. U. S. A. 102 (17), 6086–6091. 10.1073/pnas.0408452102 15837923 PMC1087912

[B130] Xu Y. Li L. Tang P. Zhang J. Zhong R. Luo J. (2023). Identifying key genes for diabetic kidney disease by bioinformatics analysis. BMC Nephrol. 24 (1), 305. 10.1186/s12882-023-03362-4 37853335 PMC10585855

[B131] Yang B. Shen F. Zhu Y. Cai H. (2024). Downregulating ANGPTL3 by miR-144-3p promoted TGF-β1-induced renal interstitial fibrosis via activating PI3K/AKT signaling pathway. Heliyon 10 (3), e24204. 10.1016/j.heliyon.2024.e24204 38322878 PMC10845249

[B132] Yang H. C. Ma L. J. Ma J. Fogo A. B. (2006). Peroxisome proliferator-activated receptor-gamma agonist is protective in podocyte injury-associated sclerosis. Kidney Int. 69 (10), 1756–1764. 10.1038/sj.ki.5000336 16598202

[B133] Yang X. Cao J. Du Y. Gong Q. Cheng Y. Su G. (2019). Angiopoietin-like protein 4 (ANGPTL4) induces retinal pigment epithelial barrier breakdown by activating signal transducer and activator of transcription 3 (STAT3): evidence from ARPE-19 cells under hypoxic condition and diabetic rats. Med. Sci. Monit. 25, 6742–6754. 10.12659/msm.915748 31494661 PMC6752095

[B134] Yang X. Cheng Y. Su G. (2018). A review of the multifunctionality of angiopoietin-like 4 in eye disease. Biosci. Rep. 38 (5). 10.1042/bsr20180557 PMC6137252 30049845

[B135] Yin W. Romeo S. Chang S. Grishin N. V. Hobbs H. H. Cohen J. C. (2009). Genetic variation in ANGPTL4 provides insights into protein processing and function. J. Biol. Chem. 284 (19), 13213–13222. 10.1074/jbc.M900553200 19270337 PMC2676053

[B136] Yoon J. C. Chickering T. W. Rosen E. D. Dussault B. Qin Y. Soukas A. (2000). Peroxisome proliferator-activated receptor gamma target gene encoding a novel angiopoietin-related protein associated with adipose differentiation. Mol. Cell. Biol. 20 (14), 5343–5349. 10.1128/mcb.20.14.5343-5349.2000 10866690 PMC85983

[B137] Zhang F. Wu P. Wang Y. Zhang M. Wang X. Wang T. (2020). Identification of significant genes with prognostic influence in clear cell renal cell carcinoma via bioinformatics analysis. Transl. Androl. Urol. 9 (2), 452–461. 10.21037/tau.2020.02.11 32420151 PMC7215011

[B138] Zhang T. Niu X. Liao L. Cho E. A. Yang H. (2013). The contributions of HIF-target genes to tumor growth in RCC. PLoS One 8 (11), e80544. 10.1371/journal.pone.0080544 24260413 PMC3832366

[B139] Zhang Y. Liu X. Zeng L. Zhao X. Chen Q. Pan Y. (2022). Exosomal protein angiopoietin-like 4 mediated radioresistance of lung cancer by inhibiting ferroptosis under hypoxic microenvironment. Br. J. Cancer 127 (10), 1760–1772. 10.1038/s41416-022-01956-7 36050447 PMC9643351

[B140] Zhao Y. Goto M. Vaziri N. D. Khazaeli M. Liu H. Farahanchi N. (2021). RNA interference targeting liver angiopoietin-like protein 3 protects from nephrotic syndrome in a rat model via amelioration of pathologic hypertriglyceridemia. J. Pharmacol. Exp. Ther. 376 (3), 428–435. 10.1124/jpet.120.000257 33443084

[B141] Zhu H. Li J. Qin W. Yang Y. He X. Wan D. (2002). Cloning of a novel gene, ANGPTL4 and the functional study in angiogenesis. Zhonghua Yi Xue Za Zhi 82 (2), 94–99. 11953136

[B142] Zhu P. Goh Y. Y. Chin H. F. Kersten S. Tan N. S. (2012). Angiopoietin-like 4: a decade of research. Biosci. Rep. 32 (3), 211–219. 10.1042/bsr20110102 22458843

[B143] Zhu P. Tan M. J. Huang R. L. Tan C. K. Chong H. C. Pal M. (2011). Angiopoietin-like 4 protein elevates the prosurvival intracellular O2(-):H2O2 ratio and confers anoikis resistance to tumors. Cancer Cell. 19 (3), 401–415. 10.1016/j.ccr.2011.01.018 21397862

[B144] Zuo Y. Dai L. Li L. Huang Y. Liu X. Liu X. (2022). ANGPTL4 regulates psoriasis via modulating hyperproliferation and inflammation of keratinocytes. Front. Pharmacol. 13, 850967. 10.3389/fphar.2022.850967 35860030 PMC9289168

[B145] Zuo Y. He Z. Chen Y. Dai L. (2023). Dual role of ANGPTL4 in inflammation. Inflamm. Res. 72 (6), 1303–1313. 10.1007/s00011-023-01753-9 37300585 PMC10256975

